# Stability of sp^3^ Carbons in Hydrogenated Graphene Quantum Dots and Their Electronic and Optical Properties Studied Using Density Functional Theory

**DOI:** 10.1021/acs.jpca.4c07825

**Published:** 2025-04-17

**Authors:** Nasiru Aminu Rano, Natalia Martsinovich

**Affiliations:** Chemistry, School of Mathematical and Physical Sciences, University of Sheffield, Sheffield S3 7HF, U.K.

## Abstract

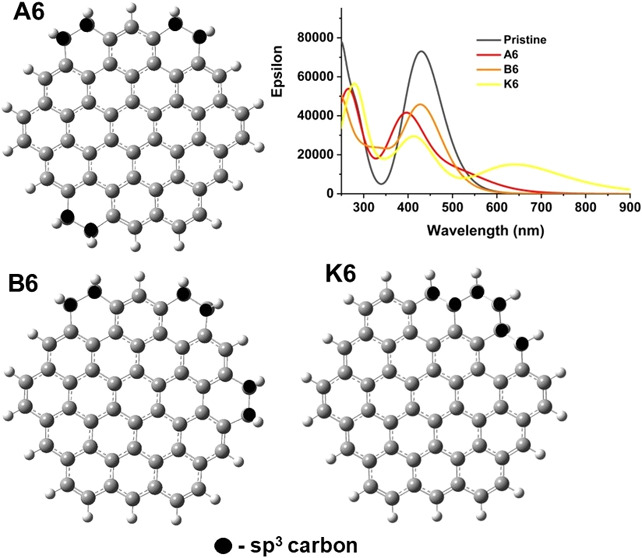

Graphene quantum dots (GQDs) are zero-dimensional nanomaterials composed of sp^2^-hybridized carbon atoms, which are widely researched because of their tunable optical properties. GQDs contain defects, such as sp^3^-hybridized carbon atoms, which may be introduced during synthesis and can affect these materials’ properties. In this study, we use hydrogenated polycyclic aromatic hydrocarbons as models for GQDs containing sp^3^-hybridized carbon atoms. We analyze the effect of sp^3^ carbons on the stabilities and electronic and optical properties of GQDs using density functional theory (DFT) and time-dependent DFT calculations. We find that sp^3^ carbons can form stable arrangements as dimers or continuous chains along the edges of GQDs. Our results reveal that the presence of sp^3^ carbons can tune the HOMO–LUMO gap, dependent on the position of sp^3^ carbons within the GQD. Calculated optical absorption spectra show a reduction in intensity and a blue shift of the main absorption peak for most of the investigated sp^3^-containing structures; additionally, the presence of sp^3^ carbons can extend the optical absorption of these structures into the red and infrared regions of the solar spectrum (600 to 900 nm), depending on the concentration and arrangement of sp^3^ carbons. These results provide insight into structural factors responsible for the variation of the electronic and optical properties of GQD nanomaterials and suggest that controlling the amount of residual sp^3^ carbon atoms introduced during synthesis can be used to tailor the properties of GQDs.

## 1 Introduction

The discovery of graphene in 2004^1^ paved the way for producing various graphene-based nanomaterials. Graphene quantum dots (GQDs) are among the latest additions to the graphene family.^2^ GQDs are zero-dimensional nanomaterials consisting of one or a few graphene layers. GQDs have great technological potential thanks to their outstanding properties, such as distinctive fluorescence arising from their nonzero band gap,^2−4^ exceptional thermal and chemical stability,^5^ biocompatibility, and nontoxicity.^4−6^ The extraordinary properties of graphene quantum dots are being explored and utilized for a variety of applications, such as energy,^2,7,8^ electronic devices,^2,4^ sensing,^3,4,7^ nanomedicine and drug delivery,^2−4,9,10^ catalysis,^2,4,7^ and water treatment.^11^ However, there are limitations that hinder the deployment of GQDs in technological applications: In particular, challenges in controlling synthesis processes lead to heterogeneity in sizes and shapes of GQD structures, which leads to variability in electronic and optical properties.^2,6^ To fully utilize the potential of GQDs, the relationship between their synthesis, structure, and properties needs to be understood.

Graphene quantum dot structures mainly comprise clusters of sp^2^ carbon atoms.^5^ However, GQDs have been shown to contain combinations of sp^2^ and sp^3^ carbons, as well as defects, dopants, and functional groups.^4,5,7^ X-ray photoemission spectroscopy (XPS) studies of GQDs show the presence of several types of carbons and functional groups, such as C=C, C–C, oxygenated carbon, and nitrous carbon.^4,12−14^ The percentage distribution of sp^2^ and sp^3^ carbons depends on the synthetic method. The top-down approach, which involves cutting larger graphitic materials, such as graphene sheets,^15,16^ carbon nanotubes,^17^ graphite oxide,^18^ etc. into small-size GQDs, produces predominantly sp^2^ carbon structures, whose π-conjugated domains determine the electronic properties of the GQD nanomaterials.^19^ The alternative approach to synthesizing GQDs is the bottom-up approach. Small sp^3^ carbon precursors, such as citric acid,^20^ amino acids,^21^ and glucose,^22,23^ can be used as starting materials to form large GQDs. GQDs obtained using the bottom-up approach contain some amount of sp^3^ carbon.^21^ Generally, the structure of GQDs may consist of a carbon core of sp^2^ and sp^3^ carbon atoms, surrounded by various functional groups, such as amino, epoxy, carbonyl, aldehyde, hydroxyl, and carboxylic acid.^7,24^ These functional groups are bonded to either the sp^2^ or sp^3^ carbons of the GQDs. This intricacy of the structures of GQDs makes it difficult to understand their structure–property relationship. Most of the experimental and computational research so far has focused mainly on investigating the effects of functional groups and dopants on the optical and electronic properties of GQDs,^5,7,24,25^ neglecting the role of sp^3^ carbon in the GQD structures. For comparison, graphene derivatives containing sp^3^ carbons, such as hydrogenated graphene (graphane), halogenated and hydroxylated graphene,^26−29^ have been studied computationally and experimentally and predicted to be semiconductors, in a qualitative change from semimetallic graphene. Partially hydrogenated or halogenated graphene materials have been synthesized, and their band gaps and conductivities have been found to depend on the extent of hydrogenation.^30−32^ By analogy, it can be expected that the presence of sp^3^-hybridized carbons in GQDs will affect their electronic properties.

To understand the effect of the carbon hybridization state on the properties of GQDs, an in-depth theoretical investigation is needed to ascertain how sp^3^ hybridized carbons affect the stability, optical, and electronic properties of GQDs. In this work, we modeled different concentrations and various possible arrangements of sp^3^ carbons, using a simple model of partially hydrogenated GQDs where the presence of hydrogen atoms converts sp^2^ hybridized carbons into sp^3^ hybridized carbons. Our results demonstrate stable arrangements of dimers and chains of sp^3^ carbons on the edges of GQDs and reveal the effect of sp^3^ carbons on the electronic and optical properties of GQDs.

## 2 Computational Method

**Figure 1:**
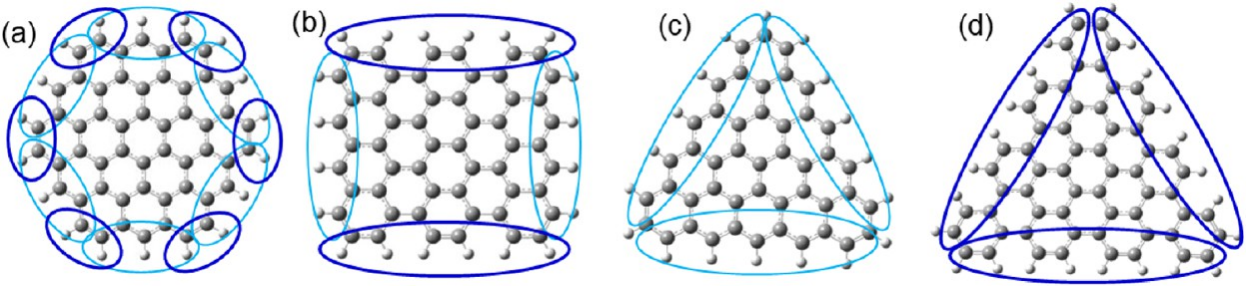
Graphene quantum dot models used in this study: (a) hexagonal C_54_H_18_ structure (circumcoronene), (b) rectangular C_54_H_20_ structure, (c) triangular C_46_H_18_ structure with zigzag edges, and (d) triangular C_60_H_24_ structure with armchair edges. Zigzag-like edges are highlighted with light-blue ovals, and armchair-like edges with dark-blue ovals.

Graphene quantum dots were modeled as polycyclic aromatic hydrocarbons (PAH) with hexagonal, rectangular, and triangular shapes shown in Figure 1. These structures contain between 46 and 60 carbon atoms and are approximately 1 nm in size, close to the experimentally synthesized small GQDs with diameters of 1–5 nm.^17,18,22^ Transmission electron microscopy studies showed that small GQDs are approximately circular in shape,^16,17,22^ while larger GQDs are polygonal.^16,23^ A hexagonal C_54_H_18_ molecule (circumcoronene, Figure 1a), which has been widely used in computational modeling of GQDs,^33−36^ was chosen as the primary computational model for GQDs because of its suitable size (1.2 nm diameter), compact near-circular shape, and presence of zigzag-like and armchair-like edges.

Density functional theory (DFT) calculations were performed using Gaussian16 software^37^ with the B3LYP functional^38^ and cc-pVTZ basis sets.^39^ The geometries of all molecules were fully optimized, and energy minima were confirmed by checking that all vibrational frequencies were positive. The B3LYP functional was chosen because it was reported in earlier studies to be highly accurate in calculations of optical absorption properties of GQDs.^40,41^ In particular, a study by Shi et al. compared several single-reference and multireference methods for calculations of excitations in PAHs, such as coronene and circumcoronene, and found that time-dependent (TD) B3LYP calculations agreed well with experimental data and with the best-performing multireference method, density functional theory/multireference configuration interaction (DFT/MRCI).^41^ To verify the accuracy of our computational method, the calculated wavelengths of the main absorption maxima of circumcoronene and of its smaller analogue, coronene, were compared with DFT/MRCI values from ref (41) and with available experimental values for coronene.^42,43^ Our B3LYP calculations accurately predicted the positions of the main absorption maxima of coronene and circumcoronene within 2 and 10 nm of the experimental and DFT/MRCI values, respectively (Table S1 in the Supporting Information (SI)). Benchmarking calculations were also carried out using the CAM-B3LYP functional,^44^ which significantly overestimated the HOMO–LUMO gap of coronene (Table S2), consistent with the results by Shi et al., who found this functional to give large errors in excitation energies.^41^

To identify the most stable spin state, all GQD structures were calculated in closed-shell singlet and open-shell triplet states. To investigate a possible biradical character of stable singlet structures, open-shell singlet states were investigated for a representative set of structures (three most stable singlet structures containing two sp^3^ carbons and five most stable singlet structures containing six sp^3^ carbons); these were found to have exactly the same ground-state energies and orbital energies as closed-shell singlets, confirming that our systems do not exhibit significant biradical behavior. This is consistent with the results for a smaller analogue, coronene, by Yeh et al.^45^ who calculated the occupation numbers of the highest occupied and lowest unoccupied natural orbitals for a series of PAHs and found coronene to have a nonradical character, which was attributed to the absence of non-Kekule structures in this molecule.

To assess the likelihood of a multireference character of circumcoronene and its derivatives, Complete Active Space Self Consistent Field calculations with two electrons and two orbitals in the active space (CASSCF(2,2)) were carried out for coronene and for the most stable circumcoronene derivatives containing two and six sp^3^ carbons (the structures that are stable in the singlet state, according to DFT). One-electron density matrix elements obtained in CASSCF(2,2) calculations confirmed that these structures are dominated by closed-shell singlet states, confirming the DFT results on the nature of the ground state.

Optical absorption calculations of all molecules in the gas phase were carried out using time-dependent density functional theory (TD-DFT), again using the B3LYP functional and cc-pVTZ basis sets by calculating 70 excitations for each molecule. Absorption spectra were generated using Gaussview software^46^ by applying Gaussian line broadening of 0.33 eV. Percentage contributions (a%) of single-particle transitions to vertical excited states were calculated using the expression bellow:^47^
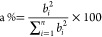
1where *b*_*i*_ are the single-particle transitions contributing to a particular excited state.

Relative energies were calculated for all sp^3^ carbon-containing GQDs of the same stoichiometries:
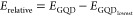
2where *E*_relative_ is the relative energy of a GQD containing sp^3^ carbons, *E*_GQD_ is the total energy of this GQD, and *E*_GQD_lowest__ is the total energy of the lowest-energy GQD with the same stoichiometry. The stoichiometries of GQDs change when sp^2^ carbons are replaced with sp^3^ carbons: for each sp^3^ carbon, one hydrogen atom is added. Therefore, the formation energy of a sp^3^-containing hydrogenated GQD, or the energy of introducing a sp^3^ carbon into a fully sp^2^ GQD, can be represented as
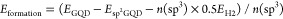
3where *E*_sp^2^GQD_ is the energy of the model fully sp^2^ GQD, *E*_H2_ is the energy of a H_2_ molecule, and *n*(sp^3^) is the number of sp^3^ carbon atoms. Here, the formation energy is calculated per sp^3^ carbon, and the energy of a H_2_ molecule is used as the standard state for hydrogen. A negative formation energy means that the presence of sp^3^ carbons is favorable compared to the fully sp^2^ GQD, while a positive formation energy means that the presence of sp^3^ carbons is unfavorable.

## 3 Results and Discussion

### 3.1 Arrangements of Single sp^3^ Carbons in a Model GQD

#### 3.1.1 Stabilities of Single-sp^3^-Containing GQDs

**Figure 2:**
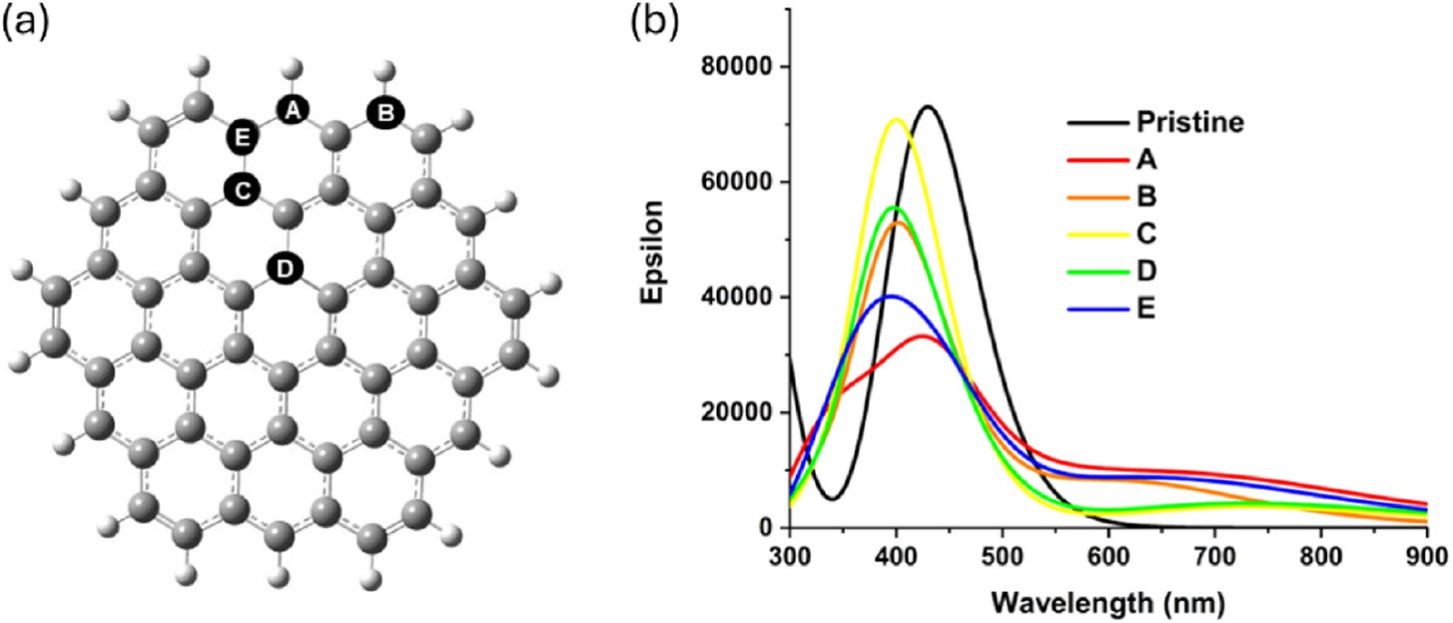
(a) Five investigated positions (A–E) for one sp^3^ carbon in the C54 GQD, ordered alphabetically from the most stable to the least stable structure. (b) Calculated optical absorption spectra of GQDs containing one sp^3^ carbon at the five different positions A–E.

A hexagonal planar C_54_H_18_ circumcoronene molecule consisting of 54 sp^2^-hybridized carbons with hydrogen-terminated zigzag-like and armchair-like edges (Figure 1a) was chosen as the primary computational model for GQDs. This structure is further termed the pristine GQD or C54 GQD. To understand the effect of sp^3^ carbons on the properties of GQDs, sp^2^ carbons in this pristine GQD were systematically converted into sp^3^ carbons at different positions at the edge and in the middle of the GQD, as shown in Figure 2a.

Comparison of relative energies of the sp^3^-containing GQDs (Table 1) shows a clear trend in stabilities dependent on the positions of the sp^3^ carbons: structures A and B with the sp^3^ carbons at the edge of the GQD are the most stable, followed by the positions C and D in the middle of the GQD, while the least stable position E has the sp^3^ carbon immediately next to the edge of the GQD. This trend obtained in B3LYP calculations is confirmed by CAM-B3LYP results presented in Table S2 in the Supporting Information (SI): the relative energies calculated using the two DFT methods agree within 0.03 eV. The observed preference for edge sites is also consistent with previous computational studies of polycyclic aromatic hydrocarbons using DFT B3LYP and multiscale multireference perturbation theory, which found edge positions to be favored in the hydrogenation of PAHs.^48,49^ The preference for hydrogenation of edge sites was attributed to low coordination numbers of those sites and their nearest-neighbor sites in the same sublattice.^49^ The stability of the edge positions can also be explained by the low distortion of these structures: optimized geometries presented in Figure S1 show that structures A and B are fully planar, while structures C-E are more distorted, with the sp^3^ carbon moving slightly out of the plane of sp^2^ carbons. Notably, the formation energies for all of these GQDs with respect to the pristine GQD and H_2_ are positive, showing that it is not thermodynamically favorable to have single sp^3^ carbons in these GQDs.

**Table 1:** Relative Energies, Formation Energies, HOMO and LUMO Energies, and Band Gaps of GQDs Containing One sp^3^ Carbon

				α**-MOs**			β**-MOs**	
structure	relative energy (eV)	formation energy (eV)	HOMO (eV)	LUMO (eV)	band gap (eV)	HOMO (eV)	LUMO (eV)	band gap (eV)
pristine			–5.18	–2.36	2.82			
A	0	0.57	–4.20	–2.33	1.87	–5.11	–3.08	2.03
B	0.27	0.83	–4.51	–2.33	2.18	–5.11	–2.80	2.31
C	1.10	1.67	–4.35	–2.33	2.02	–5.16	–3.03	2.13
D	1.12	1.68	–4.33	–2.34	1.99	–5.15	–3.08	2.07
E	1.17	1.73	–4.31	–2.34	1.97	–5.12	–3.05	2.07

#### 3.1.2 Electronic and Optical Properties of Single-sp^3^-Containing GQDs

Analysis of the highest occupied molecular orbital (HOMO) and lowest unoccupied molecular orbital (LUMO) energies of the GQDs (Table 1) shows that the HOMO–LUMO gaps of the sp^3^-containing GQDs are 0.6–0.9 eV narrower than that of the pristine GQD. Since the GQDs containing one sp^3^ carbon have one unpaired electron, the energies of both α and β electron orbitals were analyzed; similar trends and similar values were found for both α and β orbital energies. The most stable structure A has the smallest αHOMO–LUMO gap of 1.87 eV, while the next most stable structure B has a slightly larger band gap of 2.18 eV; after that, there is a weak trend of decreasing band gaps with decreased stabilities of the structures. This band gap narrowing compared to the pristine GQD can be attributed to destabilization of the singly occupied α-HOMOs, which are 0.7–1.0 eV higher than the fully occupied HOMO of the pristine GQD, and to stabilization of the β-LUMOs. CAM-B3LYP results in Table S2 show the same trend, although the band gap values are overestimated; earlier computational studies of GQDs comparing these functionals similarly found CAM-B3LYP to overestimate band gaps;^40,41^ therefore, only the B3LYP functional was used in the rest of this study.

The changes in the HOMO–LUMO gaps and orbital energies can be correlated with molecular orbital shapes shown in Figures S2 and S3 in the SI. While the β-HOMOs and the α-LUMOs of the sp^3^-containing GQDs are delocalized across the whole GQD, similarly to the HOMO and the LUMO of the unmodified pristine GQD (Figure S2), the α-HOMOs (singly occupied molecular orbitals) are mainly localized on the sp^3^ carbon and on 10–12 nearby sp^2^ carbons. In particular, when the sp^3^ carbon is in the edge position, the α-HOMO is spread primarily along the edge of the GQD. This localization is likely to destabilize the orbitals and is consistent with the α-HOMO energies being less negative compared to the HOMO of the pristine GQD, resulting in smaller band gaps. The β-LUMOs are localized in the same way, resulting in reduced β-HOMO–LUMO gaps.

These changes in the HOMO–LUMO gaps and localizations of molecular orbitals can be expected to affect the optical absorption spectra of sp^3^-containing GQDs. Calculated absorption spectra of the GQDs presented in Figure 2b show that the presence of sp^3^ hybridized carbons causes a blue shift of the main peak from 430 to 425 nm for structure A and to 395–400 nm for structures B–E. The intensity of the maximum absorption decreases, with structures A, B, and E (with the sp^3^ carbon at or near the edge) having lower maximum absorption intensities compared to structures C and D (sp^3^ carbons in the middle of the GQD). In addition, all of the sp^3^-containing structures have long-wavelength tails extending to 900 nm. Analysis of the orbitals involved in the key excitations (Table S3) shows that this long-wavelength absorption is dominated by the HOMO → LUMO transition, which was not optically active in the pristine GQD. Thus, the presence of sp^3^ carbons at a low concentration reduces the absorption in the UV and near-UV ranges but enables light absorption in the green to red region of the visible range and even in the infrared region.

### 3.2 Pairs of sp^3^ Carbons in a Model GQD

#### 3.2.1 Edge Positions of Pairs of sp^3^ Carbons in C54 GQD

##### 3.2.1.1 Stabilities of Edge Positions of sp^3^ Carbons in C54 GQDs

**Figure 3:**
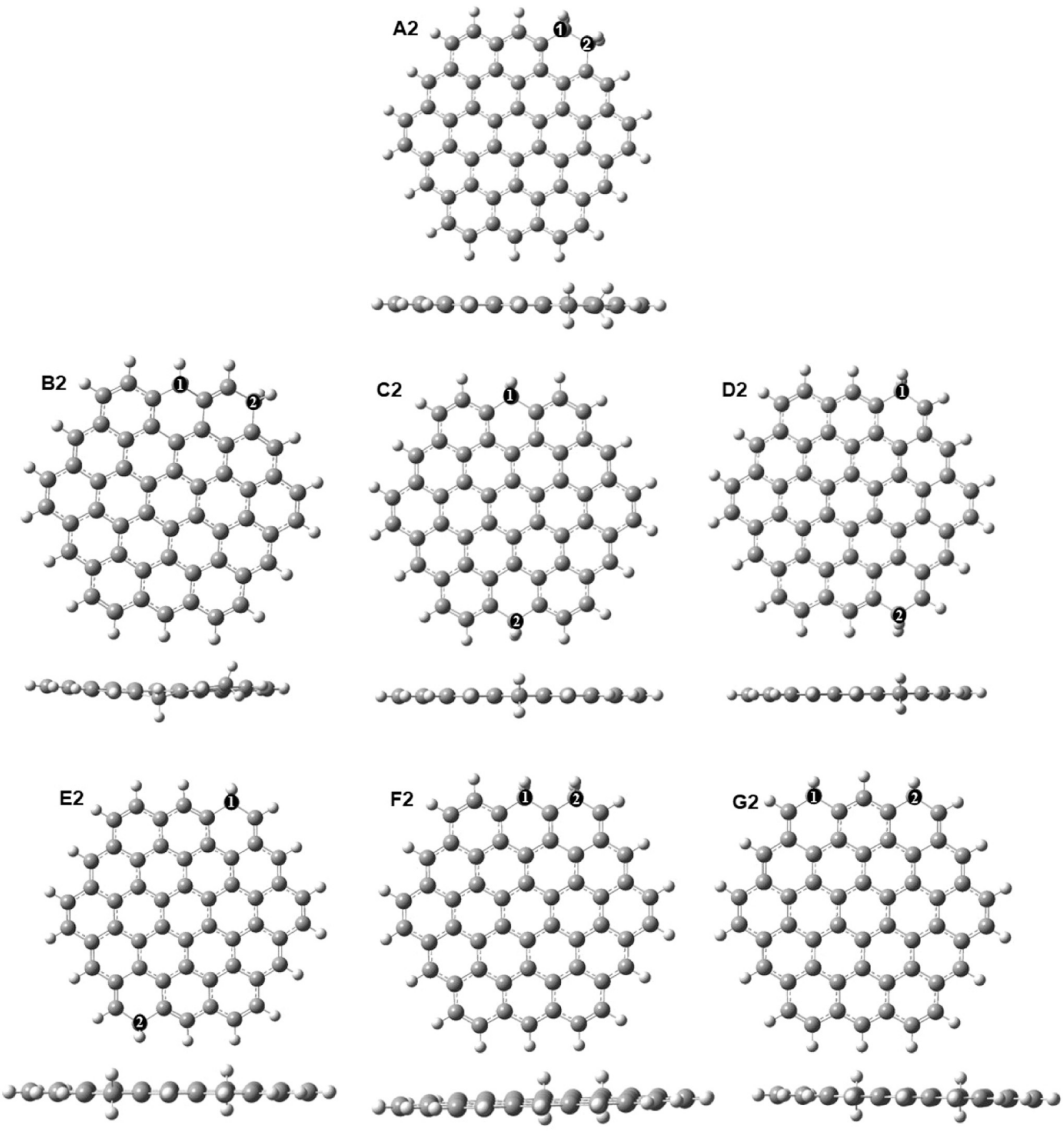
Optimized structures of GQDs containing two sp^3^ carbons at the edge positions. Geometries calculated in the singlet state are shown; the corresponding triplet geometries are the same.

To consider the effect of increasing sp^3^ carbon content and the interaction between the sp^3^ carbons, we modeled the same size GQD with multiple arrangements of pairs of sp^3^ carbons. Since the results presented in the previous section show that edge positions are preferred for sp^3^ carbons, we modeled various pairs of sp^3^ carbons at the edges of the C54 GQD, including zigzag and armchair edge positions, and nearby and distant pairs of sp^3^ carbons. The structures were calculated in both the singlet and triplet spin states. Figure 3 displays all of the investigated positions of sp^3^ carbons at the edge of the GQD (labeled A2–G2 and ordered according to their stabilities), while Table 2 shows the relative energies, formation energies, MO energies, and band gaps of the structures.

**Table 2:** Relative Energies, Formation Energies, HOMO and LUMO Energies, and Band Gaps of GQDs Containing Two sp^3^ Carbons at Edge Positions^a^

structure	relative energy (eV)	formation energy (eV/1C(sp^3^))	HOMO (eV)	LUMO (eV)	band gap (eV)
pristine			–5.18	–2.36	2.82
A2	0	–0.19	–5.02	–2.35	2.67
B2	0.82	0.22	–4.74	–2.54	2.20
C2	0.94	0.28	–4.53	–2.59	1.93
D2	1.79	0.70	–4.52	–2.65	1.87
E2	2.26 (S), 2.11 (T)	0.94 (S), 0.86 (T)	–4.16 (S), −4.24 (T)	–3.07 (S), −2.31 (T)	1.08 (S), 1.93 (T)
F2	2.69 (S), 2.02 (T)	1.15 (S), 0.82 (T)	–3.93 (S), −4.30 (T)	–3.52 (S), −2.30 (T)	0.41 (S), 2.00 (T)
G2	2.78 (S), 2.11 (T)	1.20 (S), 0.86 (T)	–3.88 (S), −4.47 (T)	–3.42 (S), −2.29 (T)	0.46 (S), 2.18 (T)

a In this table and the following tables, if the singlet (S) state is more stable, then only the values for the singlet state are reported; if the triplet (T) spin state is more stable than the singlet, then the values for both spin states are reported.

It can be seen that the most stable structure, A2, has two sp^3^ carbons directly bonded to each other. Its stability can be explained by the presence of the single σ C–C bond formed by the direct overlap of the sp^3^ hybridized orbitals. The stability decreases when the sp^3^ carbons are further apart, from the second most stable structure B2 (with the sp^3^ carbons separated by two sp^2^ carbons) to structures C2, D2, and E2, where the sp^3^ carbons are at the opposite edges of the GQDs (separated by 10 sp^2^ carbons). Among these distant pairs of sp^3^ carbons, structure C2 with the sp^3^ carbons in the middle of the zigzag edges is clearly more stable than structures D2 and E2, where the sp^3^ carbons belong to armchair-like edges. This trend is consistent with the preferred position of a single sp^3^ carbon in the middle of the zigzag edge (structure A in Figure 2a). Pairs of sp^3^ carbons separated by an odd number of sp^2^ carbons are the least stable (structures F2 and G2 with the sp^3^ carbons separated by one or three sp^2^ carbons), even though their sp^3^ carbons are placed close to each other at the same edge. Thus, clusters of sp^3^ carbons are preferred if these atoms are immediately next to each other or are in nearby positions separated by an even number of sp^2^ carbons. Interestingly, the formation energy of the most stable pair A2 is negative, showing that the presence of the nearest-neighbor pair of sp^3^ carbons is favorable compared to a fully sp^2^ GQD and a H_2_ molecule. The next two most stable sp^3^-containing structures B2 and C2 have small positive formation energies of 0.22–0.28 eV; such arrangements may be stabilized by configurational entropy, since there are multiple equivalent positions along the edge of the GQD.

##### 3.2.1.2 Electronic and Optical Properties of C54 GQDs with sp^3^ Carbons at Edge Positions

Stabilities of the sp^3^-containing GQDs correlate with their MO energies, as seen in Table 2: the HOMO energies of the singlet states become less negative, and the LUMO energies become more negative with decreasing stabilities of the structures. As a result of this systematic change in MO energies, the band gaps of the singlet structures strongly decrease as the stabilities of the structures decrease: the most stable structure A2 has a band gap of 2.67 eV, which is only 0.15 eV smaller than that of the pristine C54 GQD, while the band gap of structure F2 in the singlet state is as low as 0.41 eV. This trend can be explained by the HOMO and LUMO plots (Figure S4) showing a change in the spatial distribution of these orbitals from the most stable structure A2, where the frontier orbitals are delocalized across the whole GQD, to the less stable structure F2 in the singlet state, where the HOMO and especially the LUMO are localized around the positions of the sp^3^ carbons. Interestingly, triplet spin states become more stable than the corresponding singlets for the three least stable structures E2-G2. Figure S4 shows that the frontier orbitals for F2 in the triplet state are more delocalized than in the singlet state, which is likely to stabilize these orbitals. The HOMO energies for the triplet states are found to be more negative than for the corresponding singlets, making the triplet states more stable and resulting in their moderate band gaps of 1.9–2.2 eV.

**Figure 4:**
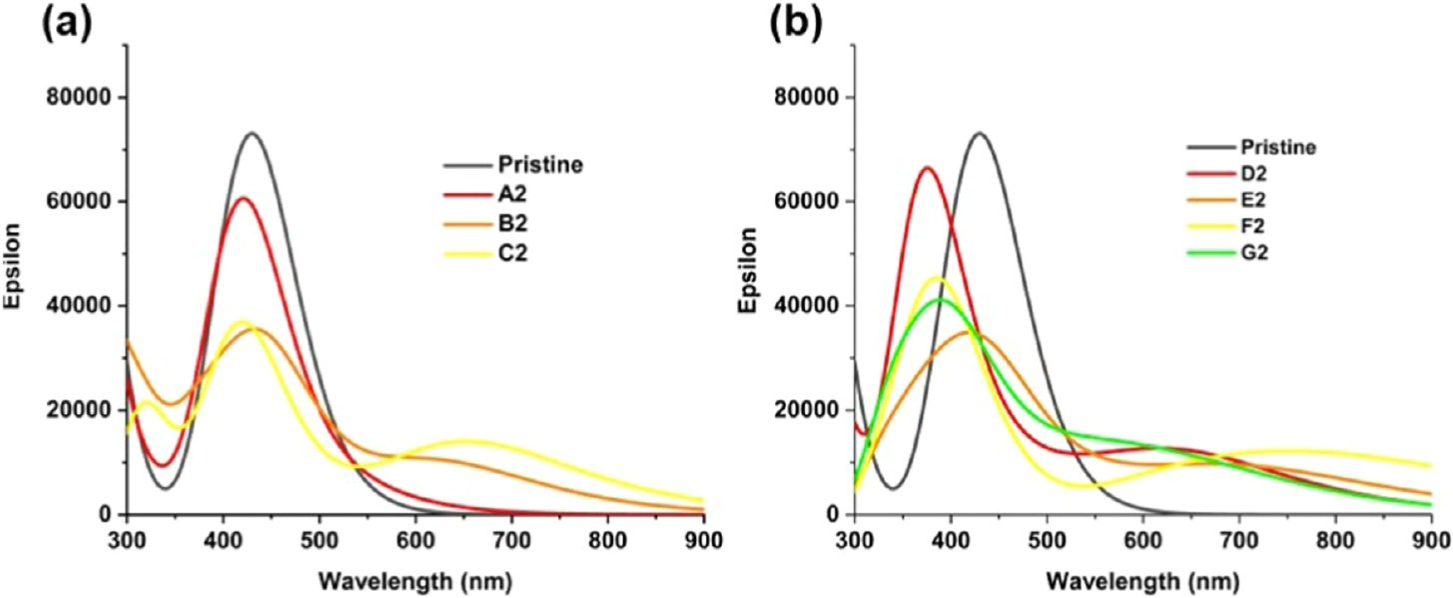
Calculated optical absorption spectra of GQDs containing pairs of sp^3^ carbons at the edge positions: (a) most stable structures A2-C2; (b) less stable structures D2-G2. Spectra of the structures in the most stable spin states are shown: singlets for A2-D2, triplets for E2-F2.

Optical absorption spectra of GQDs with pairs of sp^3^ carbons were calculated (Figure 4). These spectra show that these GQDs can be categorized into two groups according to their stabilities and light absorption properties. The three most stable structures A2, B2, and C2 have similar features in their absorption spectra. Absorption of the most stable structure A2 is almost unchanged compared to the fully sp^2^ pristine GQD, with the main absorption peak at 425 nm. The same peak but with reduced intensity is seen for the less stable structures B2 and C2, and a new peak appears at a longer wavelength of around 680 nm. Analysis of the principal transitions (Table S4) shows that the main peaks at 420–450 nm arise from combinations of transitions from HOMO–1 and HOMO to LUMO and LUMO+1, similar to the transitions responsible for the main absorption peak of the pristine C54 GQD. In contrast, the new peaks at around 680 nm in structures B2 and C2 and the small shoulder peak at 520 nm for structure A2 arise predominantly from the HOMO → LUMO transition. This transition was optically inactive in the pristine GQD but became optically active in the less symmetric sp^3^-containing GQDs. The spectra of the less stable structures D2-G2 are different from those of the pristine GQDs (Figure 4b): they show a significant blue shift of the absorption maximum from 430 nm in the pristine GQDs to 380–420 nm. This trend is the same both for the singlet and triplet states. The transitions responsible for these peaks in structures D2–G2 are complex combinations of HOMO–HOMO–3 to LUMO–LUMO+3. However, similar to the more stable structures B2–C2, these less stable structures also show absorption in the long-wavelength region of the visible range and in the infrared range, which mainly arises from the HOMO → LUMO transitions. Overall, the calculated spectra show that the presence of two sp^3^ carbons in the C54 GQD can broaden the absorption to the long-wavelength part of the visible and infrared range; however, this broad absorption is most prominent in the less stable structures B2–G2.

#### 3.2.2 Middle Positions of Pairs of sp^3^ Carbons in C54 GQD

##### 3.2.2.1 Stabilities of Middle Positions of sp^3^ Carbons in C54 GQD

**Figure 5:**
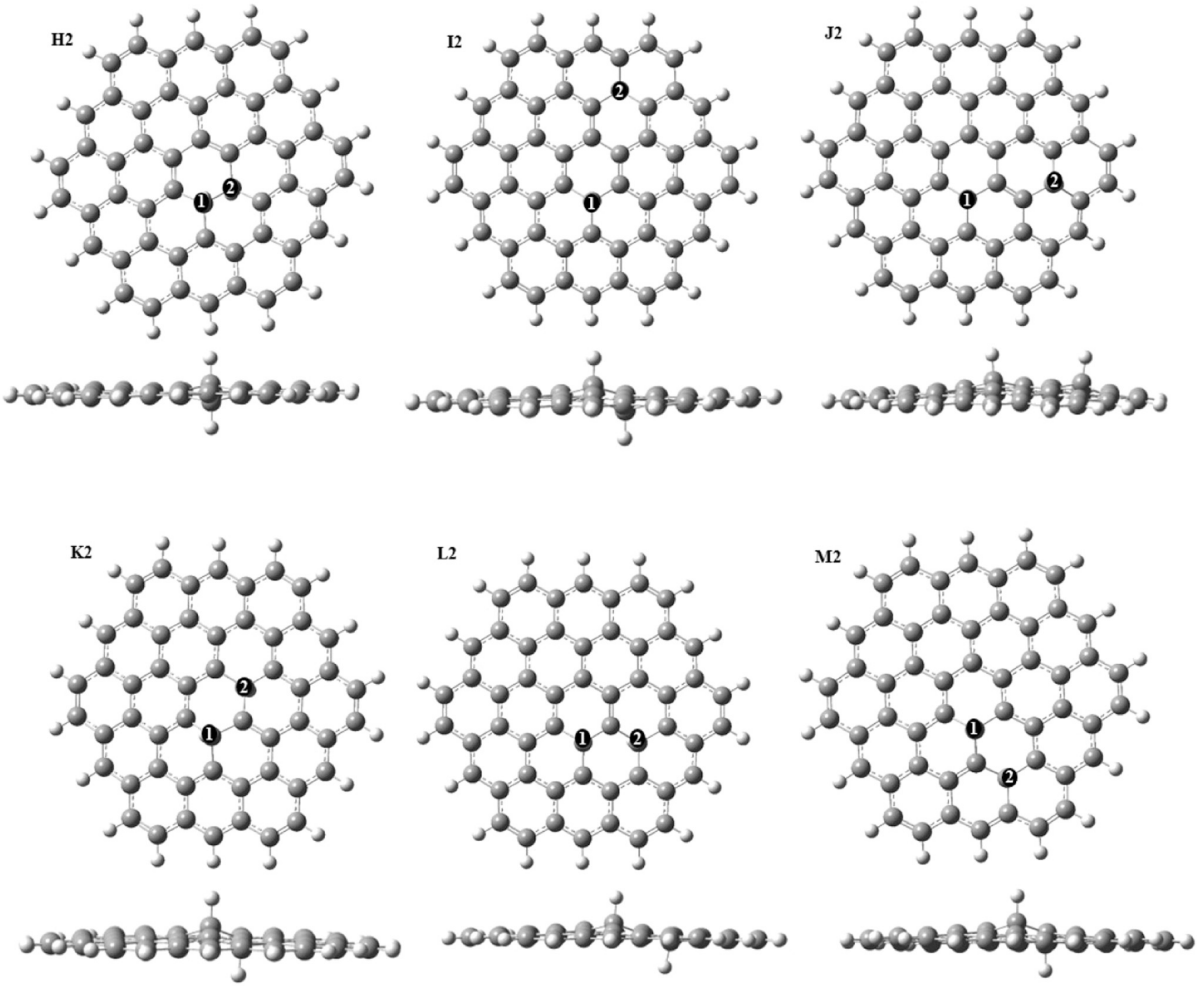
Optimized structures of GQDs containing two sp^3^ carbons at middle positions.

**Figure 6:**
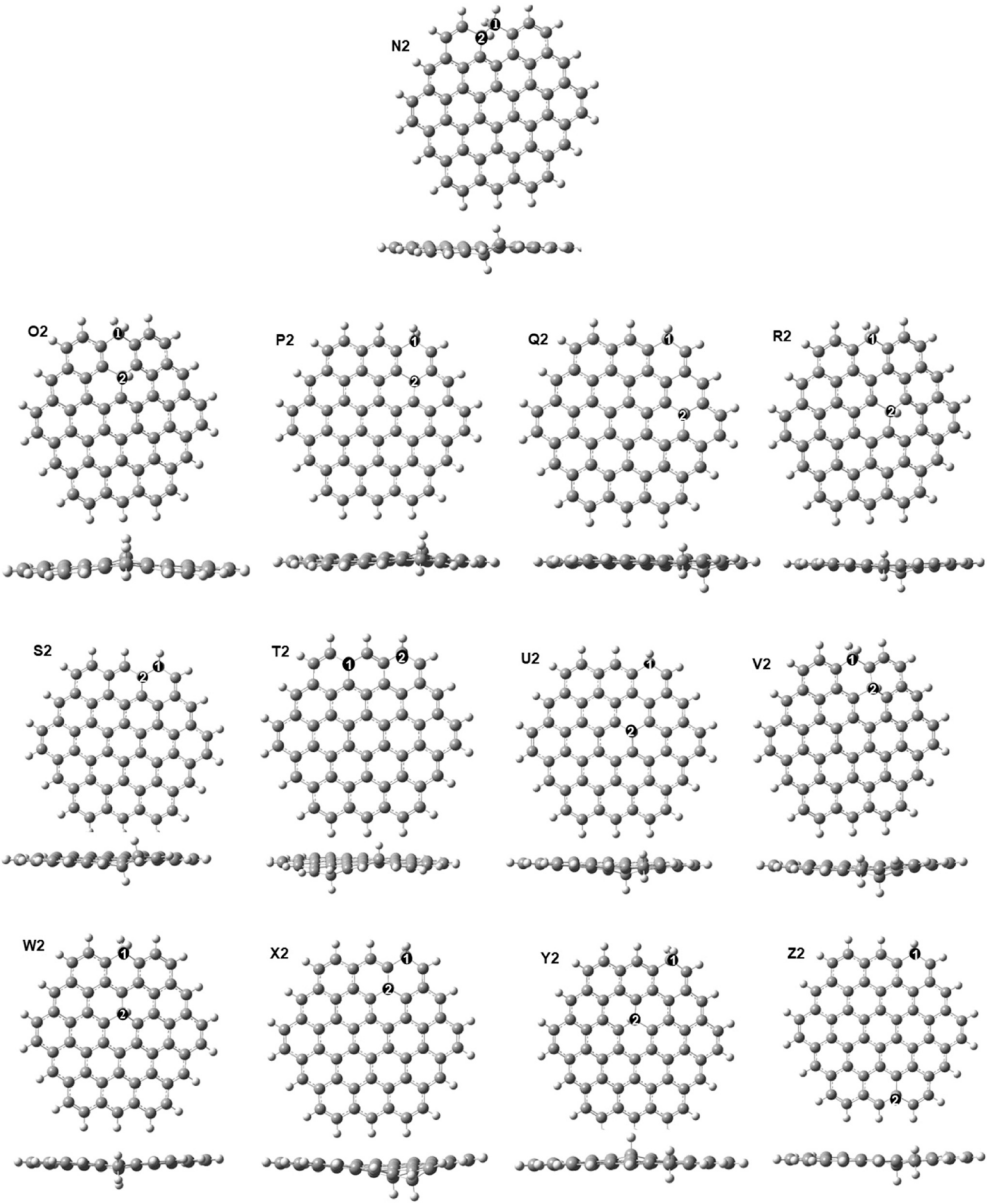
Optimized structures of GQDs containing two sp^3^ carbons at the edge and middle positions.

Since GQDs are often synthesized from sp^3^ carbon precursors, incomplete conversion of these precursors may result in some sp^3^ carbons remaining in the middle of the GQDs. Therefore, we considered several arrangements of sp^3^ carbons in the middle of the model C54 GQD (structures H2–M2, see Figure 5) and combinations of middle and edge positions of sp^3^ carbons (structures N2–Z2, see Figure 6). Table 3 shows the energies of these structures relative to those of the most stable structure A2. It can be seen that pairs of sp^3^ carbons in the middle of the GQD do not offer better stability compared to sp^3^ carbons at the edge positions: most of the middle positions are less stable than edge positions, and all of them have positive formation energies. The best of these structures is obtained when two sp^3^ carbons are bonded directly to each other in the center of the GQD (structure H2). This is consistent with the similarly directly bonded sp^3^ carbons in the all-edge structure A2 in Figure 3. Structures I2–J2 with pairs of sp^3^ carbons separated by an even number of sp^2^ carbons (two or four) are less stable than H2 by 1.2–1.5 eV, while pairs of sp^3^ carbons separated by an odd number of sp^2^ carbons (structures K2-M2) are less stable than H2 by 2.3–2.5 eV in the singlet state and by 1.9–2.0 eV in the triplet state.

**Table 3:** Relative Energies, Formation Energies, HOMO and LUMO Energies, and Band Gaps of GQDs Containing Two sp^3^ Carbons at the Middle Positions (structures H2-M2) and Combinations of Middle and Edge Positions (Structures N2-Z2)

structure	relative energy (eV)	formation energy (eV/1C(sp^3^))	HOMO (eV)	LUMO (eV)	band gap (eV)
pristine			–5.18	–2.36	2.82
middle positions of sp^3^ carbons					
H2	2.03	0.72	–4.94	–2.51	2.43
I2	3.24	1.32	–4.54	–2.62	1.91
J2	3.52	1.46	–4.72	–2.62	2.09
K2	4.34 (S), 3.93 (T)	1.87 (S), 1.67 (T)	–4.12 (S), −4.46 (T)	–3.22 (S), −2.16 (T)	0.90 (S), 2.31 (T)
L2	4.38 (S), 4.03 (T)	1.89 (S), 1.72 (T)	–4.13 (S), −4.60 (T)	–3.20 (S), −2.28 (T)	0.93 (S), 2.32 (T)
M2	4.50 (S), 3.99 (T)	1.95 (S), 1.70 (T)	–4.01 (S), −4.40 (T)	–3.34 (S), −2.31 (T)	0.68 (S), 2.09 (T)
middle and edge positions of sp^3^ carbons					
N2	0.84	0.23	–4.79	–2.59	2.20
O2	1.22	0.42	–5.05	–2.31	2.74
P2	1.68	0.65	–4.82	–2.56	2.26
Q2	1.83	0.72	–4.97	–2.33	2.65
R2	1.92	0.77	–4.59	–2.71	1.88
S2	2.00	0.81	–4.49	–2.90	1.59
T2	2.49	1.05	–4.50	–2.84	1.66
U2	2.98 (S), 2.89 (T)	1.30 (S), 1.25 (T)	–4.20 (S), −4.21 (T)	–3.13 (S), −2.26 (T)	1.07 (S), 1.95 (T)
V2	3.04 (S), 2.54 (T)	1.33 (S), 1.08 (T)	–4.02 (S), −4.34 (T)	–3.27 (S), −2.25 (T)	0.75 (S), 2.08 (T)
W2	3.11 (S), 2.65 (T)	1.36 (S), 1.13 (T)	–4.01 (S), −4.30 (T)	–3.27 (S), −2.32 (T)	0.74 (S), 1.98 (T)
X2	3.32 (S), 2.75 (T)	1.47 (S), 1.18 (T)	–4.09 (S), −4.29 (T)	–3.34 (S), −2.27 (T)	0.75 (S), 2.02 (T)
Y2	3.57 (S), 2.84 (T)	1.59 (S), 1.23 (T)	–3.87 (S), −4.33 (T)	–3.53 (S), −2.31 (T)	0.35 (S), 2.03 (T)
Z2	3.59 (S), 2.89 (T)	1.60 (S), 1.25 (T)	–3.77 (S), −4.32 (T)	–3.46 (S), −2.09 (T)	0.31 (S), 2.23 (T)

As seen in Table 3, combinations of edge and middle sp^3^ carbons can offer better stabilities than only middle positions of two sp^3^ carbons and are often comparable in stabilities to the edge positions. Structure N2 with two sp^3^ carbons bonded directly to each other is the most favorable in this group of structures, and it is the third most stable structure of all considered structures containing two sp^3^ carbons, with an energy of 0.84 eV higher than the most stable structure A2 and only 0.02 eV higher than the second most stable structure B2. Edge/middle pairs with the two sp^3^ carbons separated by two sp^2^ carbons within a single six-membered ring (structure O2 and P2) or separated by four sp^2^ carbons (structure Q2 and R2) are more stable than the all-middle positions and are comparable to the third and fourth best all-edge positions. This suggests that sp^3^ carbons may occupy combinations of edge and middle positions in GQDs. The stabilities of such structures decrease significantly when the two sp^3^ carbons are separated by an odd number of sp^2^ carbons (structures V2–Z2); these structures tend to be more stable in the triplet state than in the singlet state. Overall, this comparison of stabilities shows that nearest-neighbor pairs of sp^3^ carbons at or near the edge, such as structures A2 and N2, are the most favorable arrangements and therefore are most likely to be present in GQDs.

##### 3.2.2.2 Electronic and Optical Properties of C54 GQDs with sp^3^ Carbons at Middle Positions

The orbital energies in Table 3 show that the band gaps decrease with decreasing stabilities of sp^3^ carbon-containing GQDs, similar to the trend seen for sp^3^ carbons at the edge of the GQDs. This is caused both by destabilization of the HOMO and by stabilization of the LUMO in the singlet states. For the least stable structures in each category, the triplet spin states are more stable and have larger band gaps than their singlet state analogues, with the band gaps in the range of 1.9–2.3 eV and with little variation in the HOMO and LUMO energies. MO plots for representative structures presented in Figure S4 clarify the origin of this trend in band gaps. The most stable structure in this group, N2, has its HOMO and LUMO delocalized across the whole GQD, similar to the pristine C54 GQD and to the most stable structure with two sp^3^ carbons, A2; this results in a large band gap similar to that of the unmodified GQD. In contrast, the frontier orbitals of the less stable structures H2, K2, and Z2 are localized in the region around the sp^3^ carbons. The least stable structures, such as K2 and Z2 in the singlet state, show the strongest localization of the HOMO and the LUMO; this localization can explain the change in the orbital energies, narrowed band gaps, and low stabilities of the structures. By comparison, the frontier orbitals of structures K2 and Z2 in the triplet state are only moderately delocalized, resulting in a relatively large band gap of 2.23 eV.

**Figure 7:**
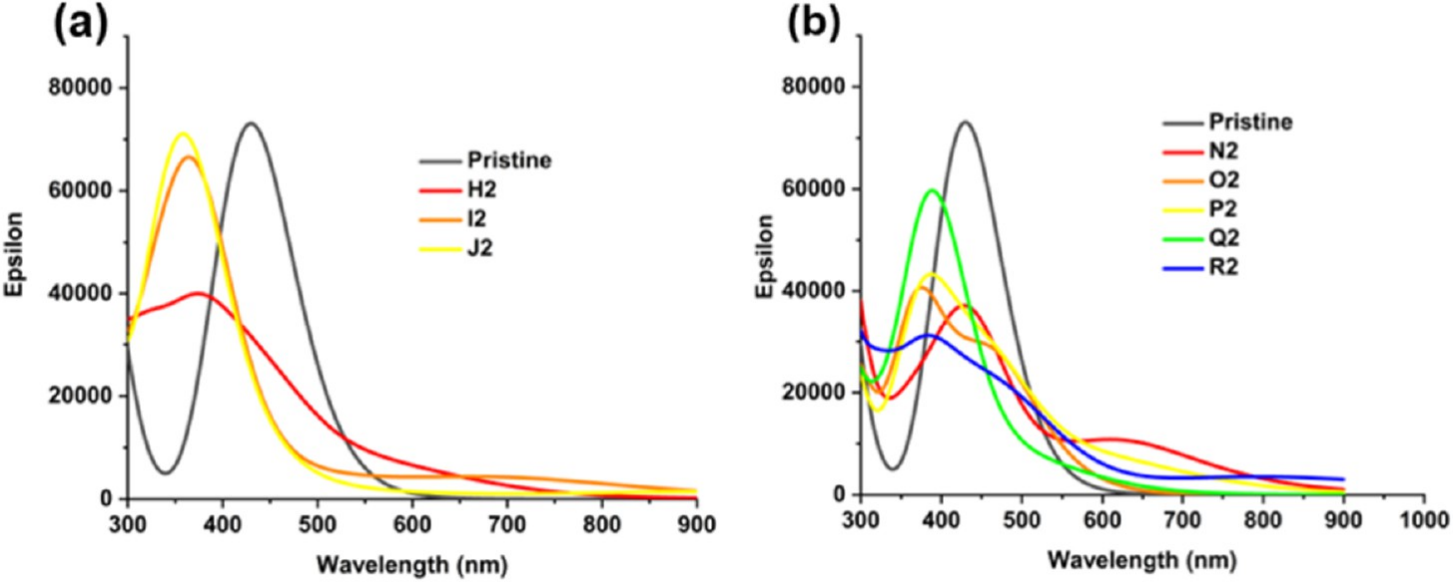
Calculated optical absorption spectra of GQDs containing pairs of sp^3^ carbons: (a) three most stable structures with two sp^3^ carbons in the middle positions, separated by 0, 2, and 4 sp^2^ carbons; (b) five most stable structures with sp^3^ carbons in the middle and edge positions, separated by 0, 2, or 4 sp^2^ carbons.

Calculated optical absorption spectra of the GQDs containing sp^3^ carbons in the middle and middle/edge positions are shown in Figure 7 (for the most stable structures) and Figure S5 (for the less stable structures). The spectra display a trend similar to that in Figure 4b (least stable edge positions): all structures display a blue shift of the main absorption peak and reduced intensities of maximum absorption compared to the pristine GQD. Additionally, most of these structures have nonzero absorption in the long-wavelength region up to 900 nm, particularly prominent in structures containing combinations of middle and edge positions of sp^3^ carbons. Analysis of the excited states of the more stable structures H2–J2 and N2-R2 (Tables S5 and S6) reveals excitations at 410–510 nm dominated by transitions from HOMO–1 and HOMO to LUMO and LUMO+1, similar to the pristine GQD, as well as strong excitations at 360–380 nm, which additionally involve higher unoccupied orbitals. Similar excitations which additionally involve lower unoccupied orbitals are observed in the less stable structures K2–M2, S2–Z2. All structures have longer-wavelength excitations (λ > 500 nm) arising from the HOMO → LUMO transition, which was inactive in the pristine GQD. Overall, it is clear from the calculated spectra in Figures 4, 7, and S5 that the presence of sp^3^ carbons causes changes in optical absorption spectra of GQDs, with the shift of the main absorption peak to the violet region of the solar spectrum and additional weaker absorption in the long-wavelength region of the visible spectrum; this may enable sp^3^-containing GQDs to harvest light in a broad range across the visible spectrum.

**Figure 8:**
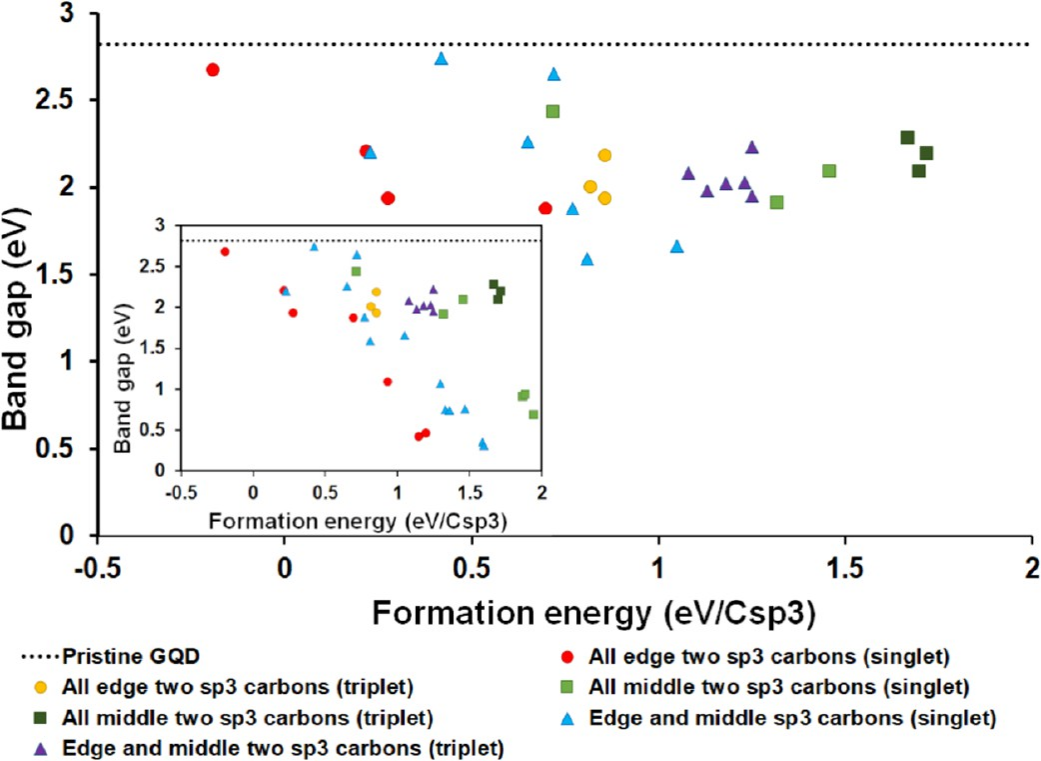
Correlation of band gaps and formation energies for GQDs containing pairs of sp^3^ carbons. The main figure shows the energies for the most stable spin states for structures A2–Z2; the inset shows the full data, including the less single singlets.

To summarize the trends in stabilities and electronic properties of sp^3^-containing GQDs, these structures’ relative energies and band gaps were compared in Figure 8. It can be seen that the arrangements of sp^3^ carbons on the edge of the GQDs are systematically more stable, while sp^3^ carbons in the middle of the GQDs are the least stable and combinations of edge and middle positions have intermediate stabilities. An inverse correlation can be seen between the energies and band gaps of the structures, where less stable structures have smaller band gaps. All of the sp^3^-containing structures have band gaps below that of the pristine G54 GQD, with band gaps of most of the structures lying between 1.5 and 2.5 eV. The systems with very small band gaps have high formation energies of ≥1 eV per sp^3^ carbon, and are unlikely to be stable (indeed, these singlet states are less stable and have smaller band gaps than the corresponding triplets); therefore very low band gaps are unlikely to be achievable by introducing pairs of sp^3^ carbons. However, there are several structures with formation energies of ≤0.5 eV per sp^3^ carbon and even one structure (A2) with a negative formation energy relative to the C54 GQD and gaseous H_2_. The five most stable sp^3^-containing structures have band gaps of 2.2–2.7 eV, which are slightly narrower than 2.82 eV in the pristine C54 GQD and correspond to the green and blue regions of the solar spectrum. Thus, the presence of sp^3^ carbons in GQDs at this low concentration is energetically feasible and likely to enable tuning (narrowing) of the band gaps to achieve light absorption in the visible range.

### 3.3 Multiple sp^3^ Carbons in Model GQDs

#### 3.3.1 Multiple sp^3^ Carbons in the Hexagonal C54 GQD

##### 3.3.1.1 Stabilities of Multiple sp^3^ Carbons in the C54 GQD

**Figure 9:**
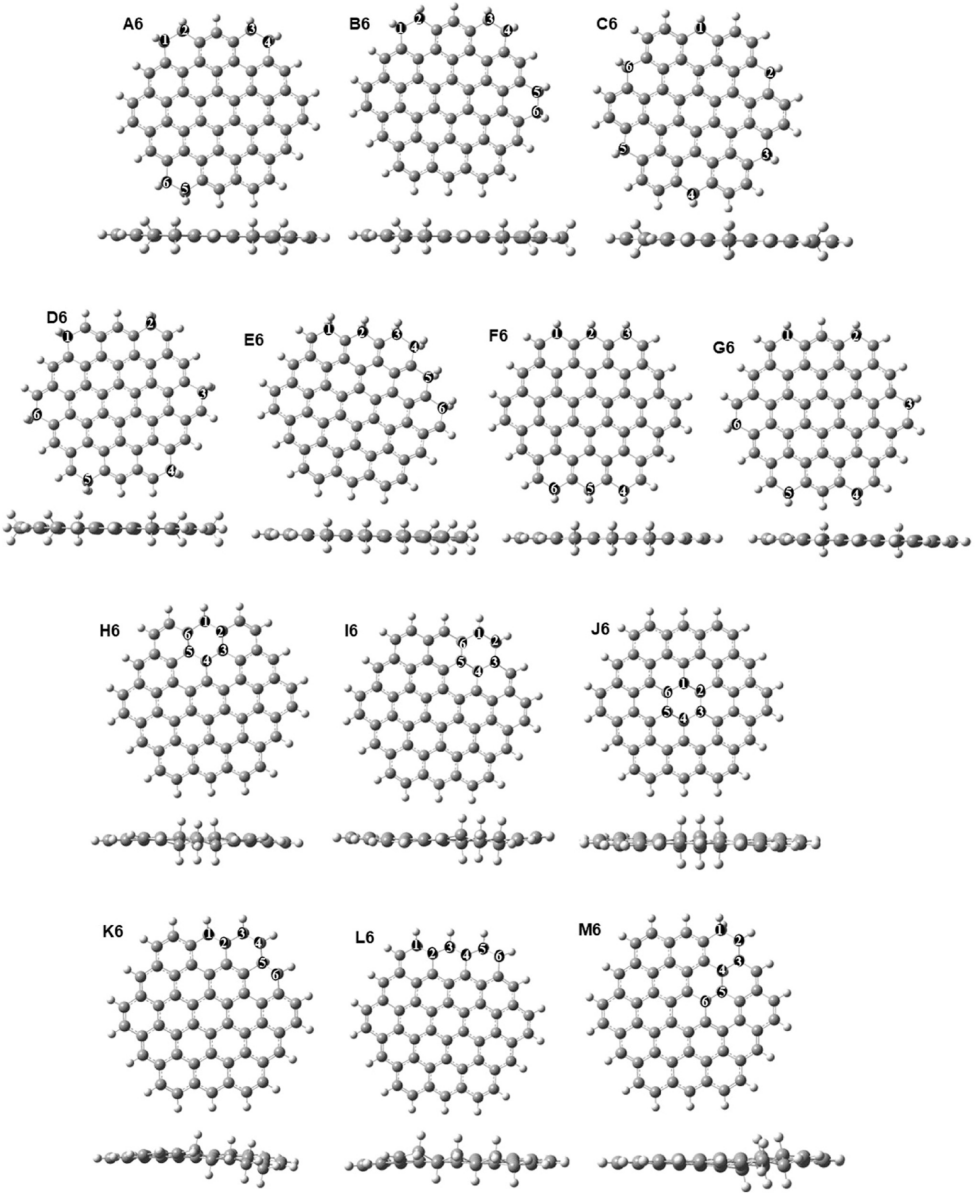
Optimized structures of GQDs containing six sp^3^ carbons in different arrangements: A6 and B6: sp^3^ dimers along the edge; C6–G6: isolated sp^3^ carbons along the edge, H6–J6: six-membered rings of sp^3^ carbons; K6–M6: chain arrangements of sp^3^ carbons.

Large-scale functionalization of GQDs is attractive for multiple applications, such as optoelectronics and magnetic materials.^50^ To progress from small-scale to large-scale functionalization, we considered GQDs containing six sp^3^ carbons, which comprise 11% sp^3^ carbons in the C54 GQD. Based on the insights obtained from our modeling of pairs of sp^3^ carbons, we considered the types of arrangements that are expected to be favorable: edge positions and clusters/chains of sp^3^ carbons. We compared several patterns of arrangement of sp^3^ carbons: (i) dimers and (ii) single sp^3^ carbons distributed across the edge of the GQDs, (iii) six-membered rings of sp^3^ carbons, and (iv) consecutive chains of sp^3^ carbons. Figure 9 shows the optimized structures of GQDs with various arrangements of six sp^3^ carbons, with the energies presented in Table 4 and the MOs of representative structures presented in Figure S6.

**Table 4:** Relative Energies, Formation Energies, HOMO and LUMO Energies, and Band Gaps of the GQD Structures Containing Six sp^3^ Carbons

structure	relative energy (eV)	formation energy (eV/1C(sp^3^))	HOMO (eV)	LUMO (eV)	band gap (eV)
pristine	–		–5.18	–2.36	2.82
(i) dimer edge arrangements					
A6	0	–0.18	–4.85	–2.17	2.67
B6	0.06	–0.17	–4.97	–2.03	2.93
(ii) isolated edge arrangements					
C6	1.12	0.00	–5.06	–1.79	3.27
D6	4.87	0.63	–4.49	–2.48	2.00
E6	5.32 (S), 4.48 (T)	0.70 (S), 0.56 (T)	–3.62 (S), −4.29 (T)	–3.35 (S), −2.85 (T)	0.27 (S), 1.44 (T)
F6	6.09 (S), 5.15 (T)	0.83 (S), 0.68 (T)	–3.54 (S), −4.19 (T)	–3.29 (S), −2.46 (T)	0.25 (S), 1.73 (T)
G6	6.14 (S), 6.45 (T)	0.84 (S), 0.89 (T)	–4.14 (S), −3.88 (T)	–2.73 (S), −2.71 (T)	1.41 (S), 1.16 (T)
(iii) six-membered ring arrangements					
H6	1.66	0.09	–5.15	–2.16	2.98
I6	1.69	0.10	–4.71	–2.56	2.15
J6	2.50	0.23	–5.47	–2.00	3.48
(iv) chain arrangements					
K6	0.14	–0.16	–4.66	–2.59	2.06
L6	0.81	–0.05	–4.50	–2.74	1.76
M6	2.36	0.21	–3.62	–3.35	1.56

As seen in Figure 9, structures A6-G6 with dimers and single sp^3^ carbons at edge positions are planar, with no geometric distortions. The most stable structures A6 and B6 (type (i) in Table 4) contain dimers of sp^3^ carbons bonded directly to one another; these structures have negative formation energies, consistent with the high stabilities of pairs of sp^3^ carbons found in the previous section, and their band gaps are similar to that of the pristine GQD. In contrast, when isolated sp^3^ carbons are spread far apart from each other along the edge of the GQD (type (ii) structures C6–G6), the stabilities and band gaps significantly decrease, both in the singlet and in the triplet state, resulting in the least stable structures considered in this section. These results support the conclusion that sp^3^ carbons prefer to be directly bonded to each other and indicate that edge positions are not necessarily stable, but dimer arrangements along the edges of GQDs are favored.

As an alternative to dimers distributed along the edge of the GQD, compact clusters of sp^3^ carbons were investigated. Structures H6, I6, and J6 (type (iii) in Table 4) contain clusters of six sp^3^ carbons arranged in hexagonal rings. In contrast to the planar structures A6–F6, these ring structures show nonplanar distortion: the sp^3^ carbons move above or below the plane of the GQDs, similar to the chair conformation of cyclohexane. It can be seen in Table 4 that the stabilities of these structures are dependent on the position of the sp^3^ ring: the hexagon of sp^3^ carbons in the middle of the GQD (structure J6) is less favorable than hexagons of sp^3^ carbons at the edges of the GQD (structures H6 and I6). Overall, such six-membered rings of sp^3^ carbons are less stable than pairs of sp^3^ carbons directed bonded to each other (structures A6-B6), but they are more stable than spread-out isolated edge sp^3^ carbon arrangements. Their stability can be attributed to the presence of single C–C bonds between sp^3^ carbons in the six-membered cyclohexane-like rings.

Another type of compact arrangement is in the form of continuous chains of sp^3^ carbons (structures K6-M6, type (iv) in Table 4). These structures display some nonplanar distortion because of the sp^3^ carbons moving out of the plane of the sp^2^ atoms. The most stable structures K6 and L6 involve six sp^3^ carbons bonded directly to each other along the edges of the GQDs. These two structures have negative formation energies and are only 0.14–0.81 eV less stable than the most stable arrangement A6. By comparison, a chain arrangement extending from the edge into the middle of the GQDs (M6) is less stable, with stability comparable to six-membered ring structures. Overall, the energies presented in Table 4 show that the most stable arrangements are those where sp^3^ carbons form dimers or long continuous chains along the edges of the GQDs. In contrast, isolated arrangements of sp^3^ carbons and clusters or chains extending into the middle of the GQDs are unfavorable.

##### 3.3.1.2 Electronic and Optical Properties of C54 GQDs Containing Multiple sp^3^ Carbons

**Figure 10:**
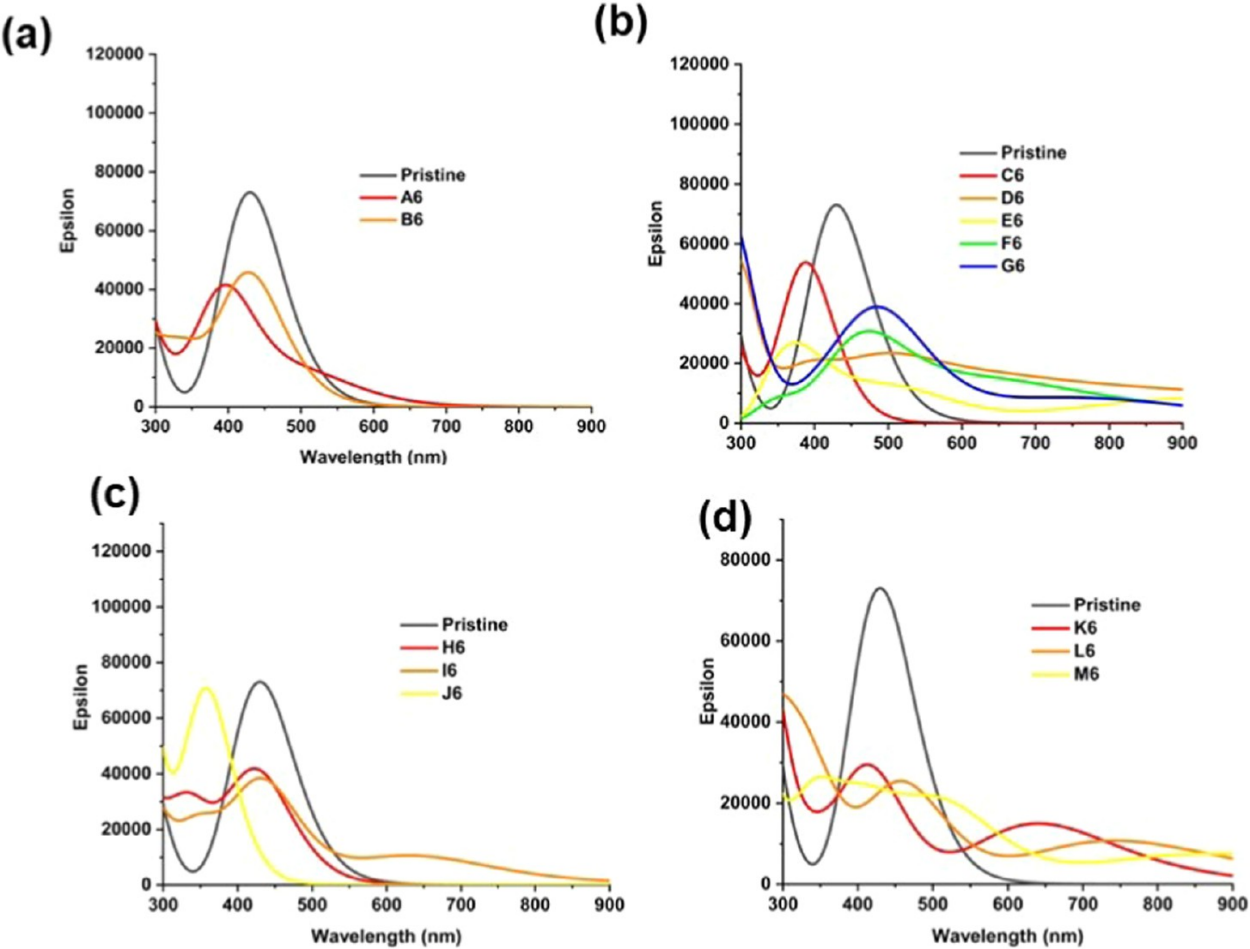
Calculated optical absorption spectra of GQDs containing six sp^3^ carbons: (a) dimer edge arrangements, (b) isolated edge arrangements, (c) six-membered ring arrangements, and (d) chain arrangements.

Calculated optical absorption spectra of the GQDs containing six sp^3^ carbons are compared in Figure 10, with the principal transitions summarized in Table S7. For most of the structures, the maximum absorption peaks are lower in intensity and are shifted compared to the pristine GQD spectrum, with the amount and nature of changes being dependent on the arrangement of the sp^3^ carbons in the structures. The absorption maxima of the three most stable edge arrangements, A6, B6, and C6, are only slightly shifted to shorter wavelengths of around 400–420 nm from the 429 nm maximum absorption peak of the pristine C54 GQD. In contrast, the less favorable edge structures D6–G6 exhibit redshifts with a tail extending across the whole visible region to the infrared region below 900 nm, in addition to peaks in the ultraviolet region. The positions of the absorption maxima for the ring arrangements H6 and I6 are almost unchanged compared to the pristine GQD; this suggests that the π system is not strongly disrupted by the formation of the sp^3^ six-membered ring at the edge, as illustrated by the MO plots in Figure S6. By comparison, the absorption maximum of structure J6 is shifted strongly to shorter wavelengths, suggesting changes in the MOs involved in the absorption. Indeed, Figure S6 shows that the HOMO and LUMO of structure J6 are doughnut-shaped, with the void at the position of the sp^3^ ring, confirming that the sp^3^ region in the middle of the sp^2^ GQD strongly disrupts the π-system. Finally, the structures with chain arrangements of six sp^3^ carbons show broad absorption across the whole visible range, which arises from two broad peaks for each structure: peaks at 400–500 nm with lower intensity than the main peak of the pristine GQD, and longer-wavelength peaks below 600 nm. The long-wavelength peak is particularly pronounced in structure K6, and it arises from the HOMO → LUMO transition which is inactive in the pristine GQD.

**Figure 11:**
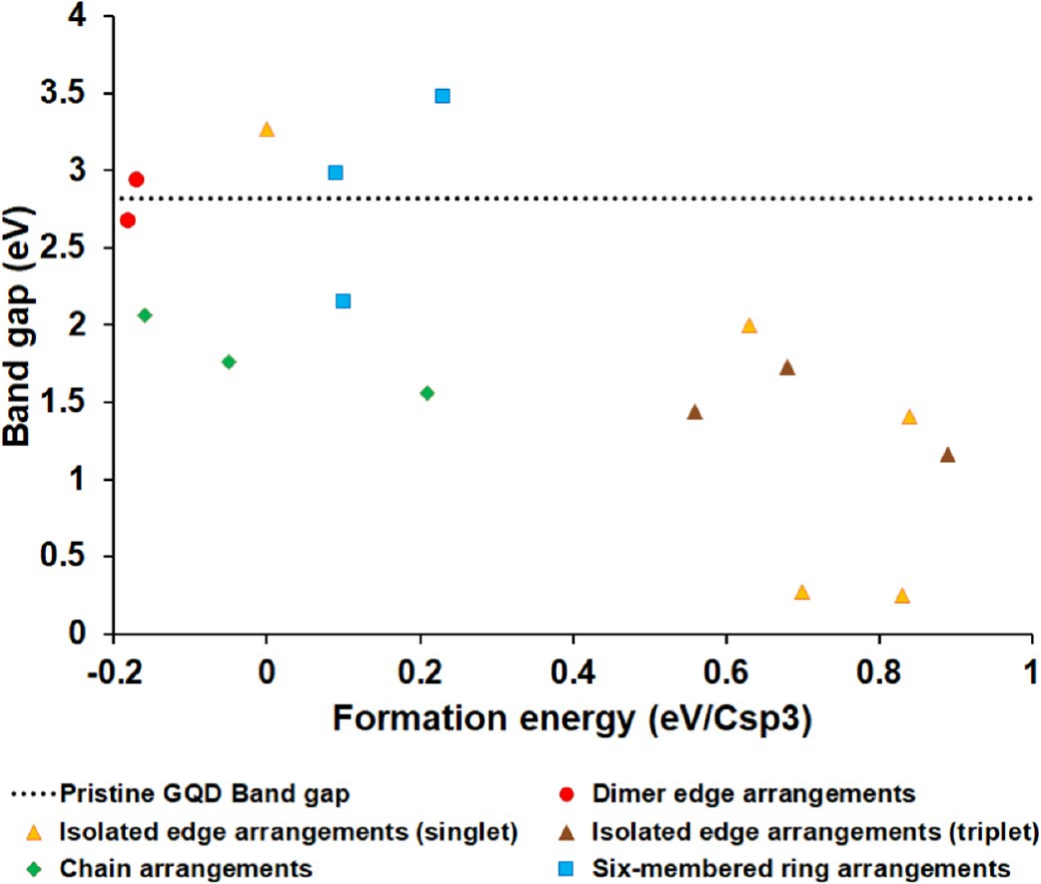
Correlation of band gaps and formation energies for GQDs containing six sp^3^ carbons.

To summarize the trends in stabilities and electronic properties of these structures, band gaps were plotted against formation energies in Figure 11 for all the studied arrangements of six sp^3^ carbons in the C54 GQD. While there is no strong correlation between stabilities and band gaps, it is clear that the narrowest band gaps are found in the least stable structures (isolated edge arrangements), which are unlikely to exist. The most stable structures with negative formation energies are the dimer edge arrangements and chain arrangements; these structures have band gaps either similar (dimer edge structures) or slightly smaller (chain structures) than the pristine GQD. Thus, dimer and chain arrangements of sp^3^ carbons in GQDs can be stable, and they enable tuning of the band gaps and optical absorption spectra of GQDs.

#### 3.3.2 Multiple sp^3^ Carbons in Alternative GQD Shapes

Since the shapes of experimentally produced GQDs are not well defined, we considered alternative GQD shapes of similar sizes to verify whether the key trends found in the previous section (the preference for clustering of sp^3^ carbons along GQD edges and the tuning of band gaps) apply universally to various GQD shapes. The alternative studied GQD shapes were as follows: a rectangular (R) C_54_H_20_ GQD containing distinct armchair and zigzag edges (Figure 1b), a triangular C_46_H_24_ GQD with zigzag edges (TZ, Figure 1c), and a triangular C_46_H_24_ GQD with armchair edges (TA, Figure 1d).

##### 3.3.2.1 Sp^3^ Carbons in the Rectangular GQD with Armchair and Zigzag Edges

**Figure 12:**
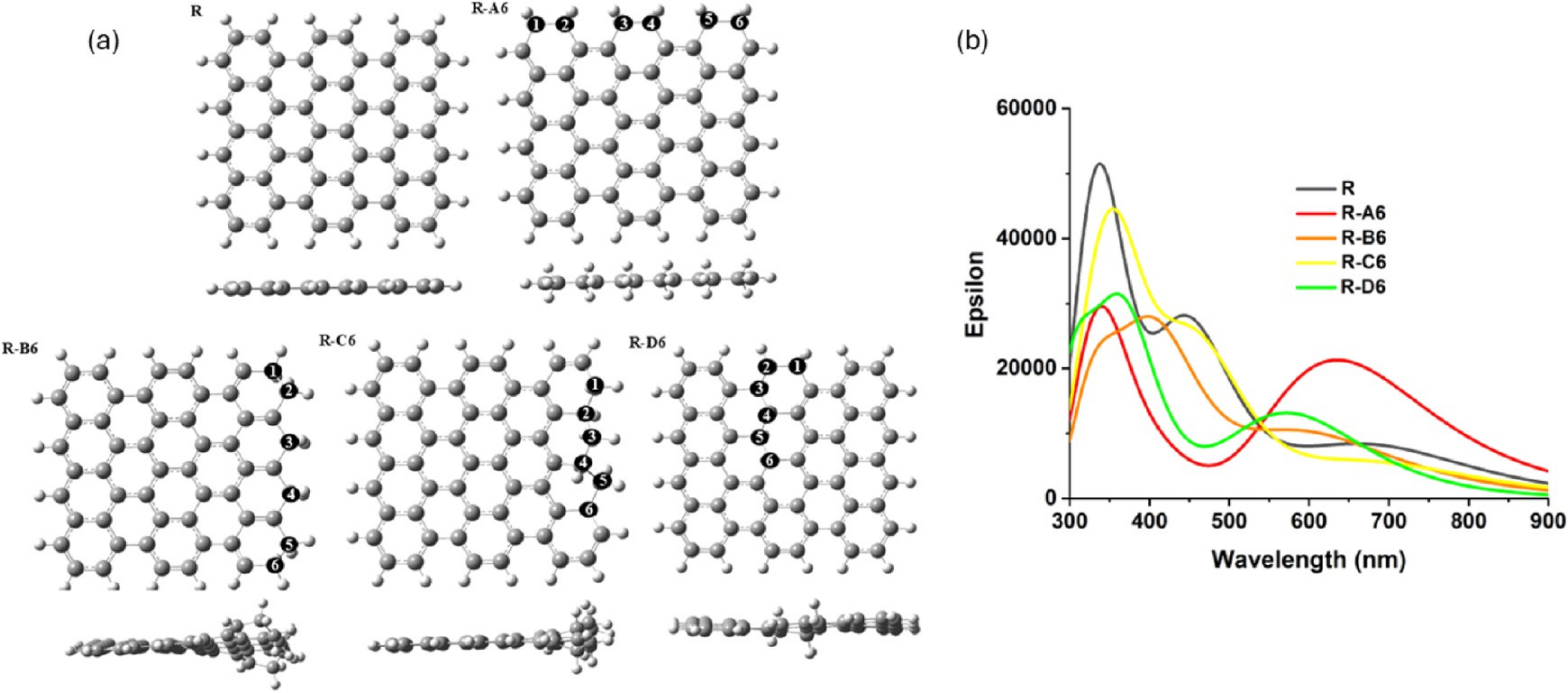
(a) Optimized structures of rectangular GQDs: R: pristine fully sp^2^ GQD; R-A6 to R-D6: GQDs containing different arrangements of six sp^3^ carbons. R-A6: dimers of sp^3^ carbons along the armchair edge; R-B6: chain of sp^3^ carbons along the zigzag edge; R-C6: continuous chain of edge and near-edge sp^3^ carbons along the zigzag edge; R-D6: continuous chain extending from the armchair edge into the middle. (b) Calculated optical absorption spectra of rectangular GQDs with and without sp^3^ carbons.

To investigate the preference of sp^3^ carbons toward either armchair or zigzag edges, hydrogenation of the rectangular GQD was investigated, as shown in Figure 12a. The energies shown in Table 5 show that dimers of sp^3^ carbons placed along the armchair edge (structure R-A6) are the most stable, followed by a chain extending from the armchair edge into the middle of the GQD (structure R-D6). By comparison, structures that contain sp^3^ carbons along the zigzag edge (R-B6 with a chain of single sp^3^ carbons along the zigzag edge, and R-C6 with a continuous chain of edge and near-edge sp^3^ carbons along the zigzag edge) are less stable. The stabilities of the armchair edge arrangements can be attributed to the presence of σ-bonded pairs of carbons along the armchair edge, similar to the favored structure A2 in the C54 GQD. Zigzag edge positions do not offer such stable pairs: zigzag arrangements can be either chains of isolated sp^3^ carbons separated by sp^2^ carbons (structure R-B6 with the pattern similar to the unstable structure F2 in Figure 3) or continuous chains involving edge and near-edge positions (structure R-C6 with the pattern similar to structure N2 in Figure 6). However, the formation energies of all these sp^3^-containing structures relative to the pristine rectangular GQD and H_2_ molecules are close to zero, suggesting that such structures may exist under realistic conditions, especially when entropically stabilized.

**Table 5:** Relative Energies, Formation Energies, HOMO and LUMO Energies, and Band Gaps of Rectangular GQDs Structures: Fully sp^2^ Structure R, and Structures R-A6 to R-D6 Containing Six sp^3^ Carbons at Various Edge Positions^a^

structure	relative energy (eV)	formation energy (eV/1C(sp^3^))	αHOMO, βHOMO (eV)	αLUMO, βLUMO (eV)	αband gap, βband gap (eV)
R (T)			–4.53, −5.10	–2.39, −3.03	2.14, 2.07
R-A6 (T)	0.00	0.00	–4.60, −4.47	–2.58, −2.55	2.03, 1.92
R-B6 (T)	0.37	0.06	–4.48, −4.94	–2.28, −2.91	2.20, 2.03
R-C6 (T)	0.77	0.13	–4.34, −4.87	–2.10, −2.78	2.24, 2.09
R-D6 (T)	0.14	0.02	–4.48, −5.33	–1.96, −2.93	2.52, 2.40

a The stable spin states for all of these structures are triplets.

The trends in the electronic properties of the rectangular sp^3^-containing GQDs are somewhat different from those of the hexagonal GQD model. All rectangular structures studied in this work are more stable in the triplet state than as singlets. Interestingly, the band gaps of the sp^3^-containing GQDs are not very different from the pristine rectangular GQD. The exception is structure R-D6, where the band gap increased; this can be attributed to quantum confinement, because the chain of sp^3^ carbons effectively divides the GQD into two smaller fragments. The calculated optical absorption spectra of all rectangular GQDs in this study span the whole visible range (Figure 12b), unlike the hexagonal GQD considered in the previous sections. For GQDs containing sp^3^ carbons, the absorption is reduced in the ultraviolet and blue regions but is preserved in the red region. Notably, absorption in the red region is enhanced for the most stable structure R-A6, suggesting that, as with hexagonal GQDs, the presence of sp^3^ carbons can be beneficial for light harvesting.

##### 3.3.2.2 Sp^3^ Carbons in Triangular GQDs with Armchair or Zigzag Edges

**Figure 13:**
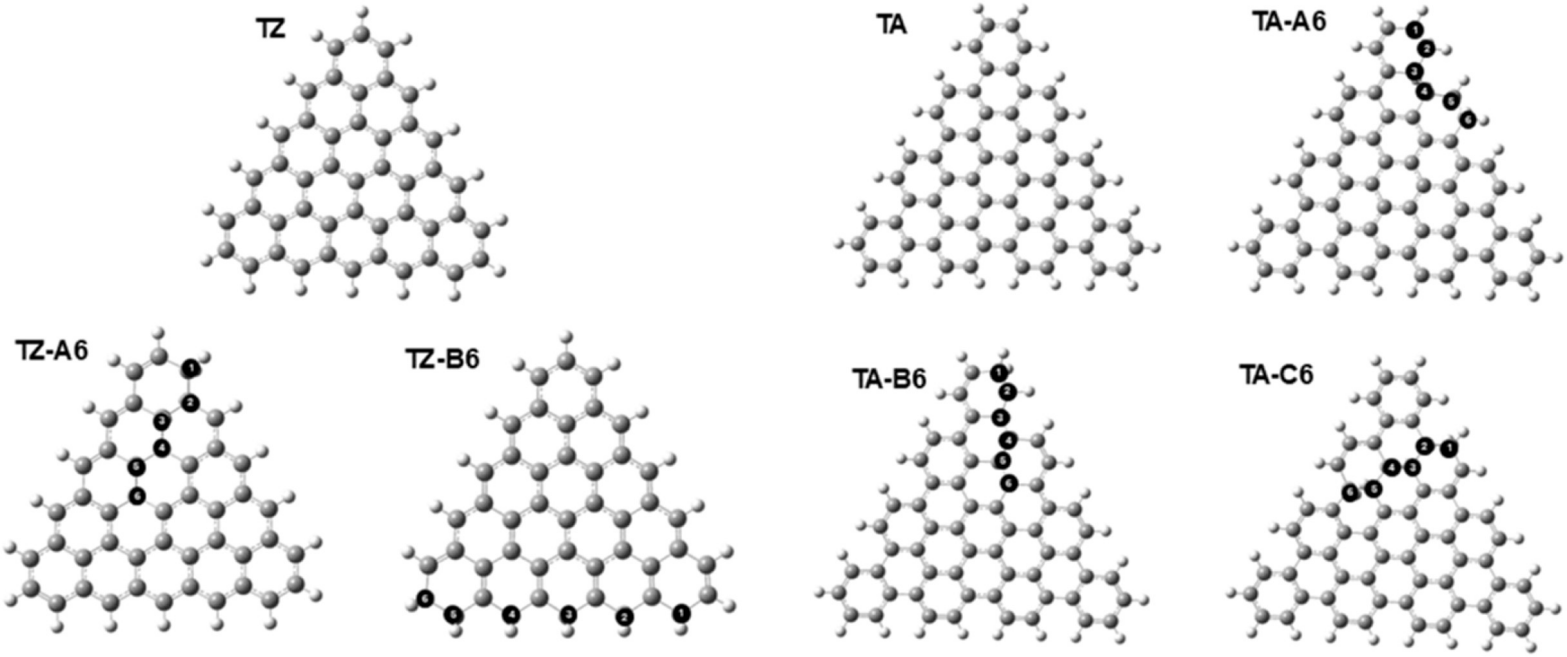
Optimized structures of triangular GQDs without and with sp^3^ carbons. TZ: triangular GQD model with zigzag edges; TZ-A6: six sp^3^ carbons extending from the edge into the middle of the GQD; TZ-B6: six sp^3^ carbons along the zigzag edge. TA: triangular GQD model with armchair edges; TA-A6: six sp^3^ carbons along the armchair edge; TA-B6: six sp^3^ carbons extending from the edge into the middle of the GQD; TA-C6: six sp^3^ carbons in the middle of the GQD.

To investigate the effect of the shape of the GQDs as well as the type of edge, incorporation of sp^3^ carbons into triangular GQD models containing only zigzag edges (labeled TZ) or only armchair edges (labeled TA) was studied. Using insights on stable structures from the previous sections, sp^3^ carbons were placed either along the zigzag or armchair edge, or as chains extended from the edge into the middle of the GQD (Figure 13 and energies in Table S8). In the model with zigzag edges, incorporation of sp^3^ carbons either in the middle of the GQD or as a chain of isolated atoms along the edge is unfavorable, according to the energies presented in Table S8. The HOMO–LUMO gaps are slightly increased upon incorporation of sp^3^ carbons, but the effect on optical properties is not uniform: optical absorption of structure TZ-A6 (sp^3^ carbons along the edge) is extended into the visible range, but absorption of structure TZ-B6 (sp^3^ carbons in the middle) is constrained to the violet and ultraviolet range (Figure S7).

In the triangular GQD model with armchair edges, incorporation of sp^3^ carbons in a continuous chain along the armchair edge is favorable (structure TA–A6 in Figure 13), while chains of sp^3^ carbons extending into the middle of the GQD (structures TA–B6 and TA–C6) are significantly unfavorable, as shown by the energies in Table S8. The stability of the armchair edge positions and the instability of the middle chain positions are consistent with the results obtained for the hexagonal and rectangular structures. In contrast, the electronic properties of the TA-based GQDs show a distinct trend: the band gaps of all the sp^3^-containing structures are significantly decreased, especially for the most stable structure TA–A6. Therefore, this arrangement of sp^3^ carbons along the armchair edges of the triangular GQD offers the best opportunity for tuning the optical absorption properties of the GQDs.

This comparison of the properties of differently shaped GQDs enables us to highlight some general trends in stabilities and electronic properties of sp^3^-containing GQDs. The best stabilities are achieved if sp^3^ carbons are placed along armchair edges, either as dimers or as continuous chains, in contrast to unstable positions along zigzag edges or in the middle of GQDs. Regarding electronic properties, there is no single trend of decreasing or increasing HOMO–LUMO gaps: changes in HOMO–LUMO gaps depend both on the shape of the GQD and on the position of sp^3^ carbons at the edges or in the middle of the GQD. However, there is a general trend observed in optical properties: stable arrangements of sp^3^ carbons tend to extend optical absorption into the red region of the visible spectrum or enhance absorption in this region. This broadening of optical absorption can be attributed to HOMO → LUMO transitions becoming optically active in sp^3^-containing GQDs, probably due to their reduced symmetry compared to their parent pristine GQDs.

## 4 Conclusions

**Figure 14:**
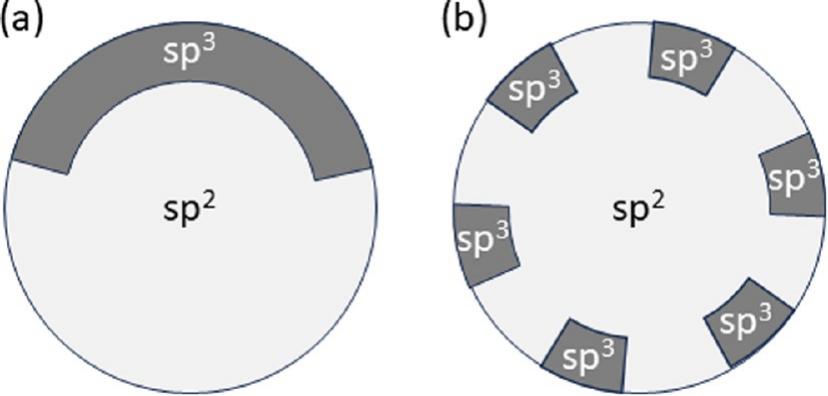
Schematic arrangement of sp^3^ carbons in a predominantly sp^2^ GQD: (a) partial rim in the form of a continuous chain of sp^3^ carbons along the armchair edge surrounding the sp^2^ core of the GQD, and (b) partial rim in the form of dimers of sp^3^ carbons surrounding the sp^2^ core of the GQD.

This study systematically investigated the effects of sp^3^ carbons on the stabilities, electronic properties, and optical absorption of graphene quantum dots, using DFT and TD-DFT approaches. By hydrogenation of model GQD structures, various positions and concentrations of sp^3^ carbons and several GQD shapes were explored. Our results show that positions of sp^3^ carbons at the edges of GQDs are preferred over positions in the middle of the GQDs. Their stabilities also depend on the arrangement of these sp^3^ carbons: clusters of sp^3^ carbons were found to be favorable, with a clear preference toward chains and dimers along armchair edges of GQDs, while zigzag edge positions, six-membered rings of sp^3^ carbons and chains extending from the edge into the middle of the GQD were less stable. Isolated sp^3^ carbons were the least stable. The most stable hydrogenated structures in this work were thermodynamically stable with respect to fully sp^2^ GQDs and H_2_ gas. These results suggest that bottom-up synthesis routes where predominantly sp^3^ carbon precursors are converted to predominantly sp^2^ graphene dots may leave residual sp^3^ carbons along the edges of the GQDs. A realistic arrangement of sp^3^ carbons in a GQD would be a rim of sp^3^ carbons (either a continuous chain or multiple dimers) along the armchair edge, surrounding the sp^2^ core of the GQD, as schematically shown in Figure 14.

Our results show that the various arrangements of sp^3^ carbons can affect the electronic and optical properties of GQDs. In particular, the presence of sp^3^ carbons typically causes a blue shift and/or a reduction in intensity of the main absorption peak of the model GQD; additionally, many sp^3^-containing structures show absorption extended into the red region of the visible spectrum. This broadening of optical absorption suggests that presence of sp^3^ defects is not detrimental to optical absorption and may be favorable for tuning light-harvesting abilities of GQDs. This research provides valuable insights into structural factors responsible for electronic properties and light absorption of GQDs. By highlighting the stability and the effects of sp^3^ carbons inherited from molecular precursors in bottom-up synthesis, it raises a prospect of controllable synthesis of GQDs with full or partial conversion of sp^3^ precursors to sp^2^ graphene quantum dots and offers a possibility of using these sp^3^ defects as a means to tailor the properties of GQDs for optoelectronic applications.

Besides sp^3^ carbon defects introduced in synthesis, various functional groups are known to be present in GQDs, such as amines, hydroxyls, or carboxylic acids. These groups are likely to be on the edges of the GQDs, and they may be attached to either sp^2^ or sp^3^ carbons. We believe that the insights from the simple model investigated in this work, such as the band gap narrowing and the preference for clustering, are also applicable to functional groups attached to sp^3^ carbons at the edges of GQDs. Future research could investigate different functional groups, such as amines or hydroxyls, bonded to sp^3^ carbons in GQDs, to distinguish the effects caused by sp^3^ carbons and by the specific functional groups.

## Data Availability

Data for this article (coordinates of structures and optical absorption calculations results in the Gaussian.log format) are available at the University of Sheffield repository ORDA at https://figshare.com/s/893a280d237cfdb15686.

## Supporting Information Available

Results of test calculations using the CAM-B3LYP functional (formation energies and orbital energies); HOMO and LUMO images of the pristine GQD and of GQDs containing 1, 2, and 6 sp^3^ carbons; analysis of the principal excited states of the sp^3^-containing GQDs; and stabilities of alternative sp^3^-containing rectangular GQDs (PDF)
