# Supplementary material for: Stability of sp3 Carbons in Hydrogenated Graphene Quantum Dots and Their Electronic and Optical Properties Studied Using Density Functional Theory
Source: J Phys Chem A. 2025 Apr 17;129(17):3790–806. doi: 10.1021/acs.jpca.4c07825 (PMC12051202; doi:10.1021/acs.jpca.4c07825)
Supplement: Supplementary file 1 — jp4c07825_si_001.pdf [file jp4c07825_si_001.pdf]

Supporting Information for

Stability of sp^3 Carbons in Hydrogenated Graphene Quantum Dots and Their Electronic and Optical Properties Studied Using Density Functional Theory

Nasiru Aminu Rano[a] and Natalia Martsinovich[a]*

[a]Chemistry, School of Mathematical and Physical Sciences, University of Sheffield, Sheffield, S3 7HF, UK

*E-mail: n.martsinovich@sheffield.ac.uk

Table S1: Maximum absorption peaks for $C_{24}H_{12}$ and $C_{54}H_{18}$ obtained in this work (DFT B3LYP method), compared to literature values calculated using density functional theory/multireference configuration interaction (DFT/MRCI) method and experimental UV-Vis spectroscopy measurements.

Structure	Calculated value, nm (This work)	Literature value, nm (Theory)	Literature value, nm (Experiment)
$C_{24}H_{12}$	302	302 (DFT/MRCI) [1]	300 [2], 305 [3]
$C_{54}H_{18}$	430	440 (DFT/MRCI) [1]	-

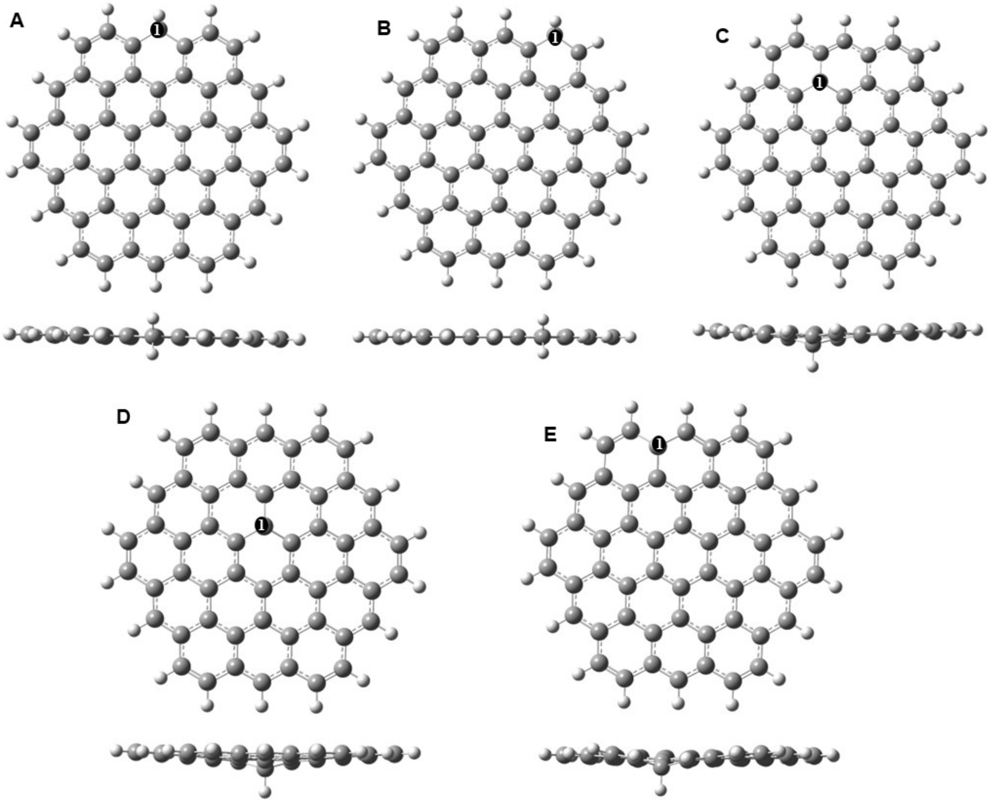

Figure S1: Optimized structures of the five investigated positions (A, B, C, D and E) for one sp^3 carbon in the C54 GQD, ordered alphabetically from the most stable to the least stable structure.

Table S2: Relative energies, formation energies, HOMO and LUMO energies and band gaps of GQDs containing one sp^3 carbon calculated using the CAM-B3LYP functional.

			**α-MOs**			**β-MOs**		
Structure	Relative energy (eV)	Formation energy (eV)	HOMO (eV)	LUMO (eV)	Band gap (eV)	HOMO (eV)	LUMO (eV)	Band gap (eV)
Pristine	-		-6.14	-1.45	4.69	-	-	-
A	0	0.46	-5.21	-2.33	3.82	-6.10	-2.12	3.98
B	0.26	0.72	-5.58	-2.33	4.19	-6.08	-1.79	4.30
C	1.13	1.59	-5.46	-2.33	4.08	-6.16	-1.98	4.18
D	1.13	1.59	-5.41	-2.34	4.04	-6.18	-2.05	4.13
E	1.16	1.62	-5.35	-2.34	3.96	-6.11	-2.06	4.05

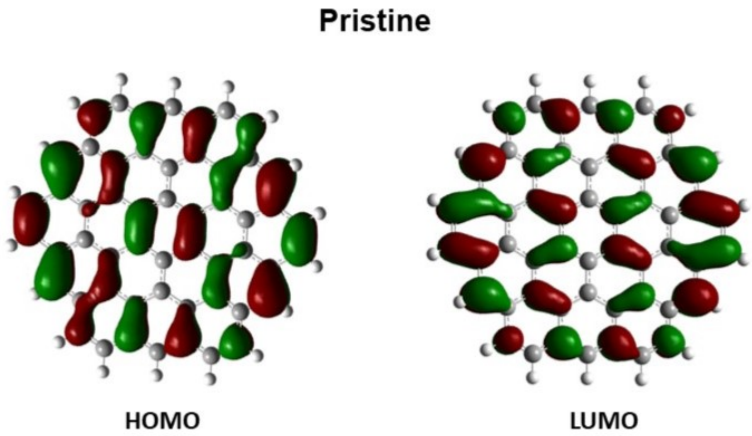

Figure S2: HOMO and LUMO of the pristine $C_{54}H_{18}$ GQD.

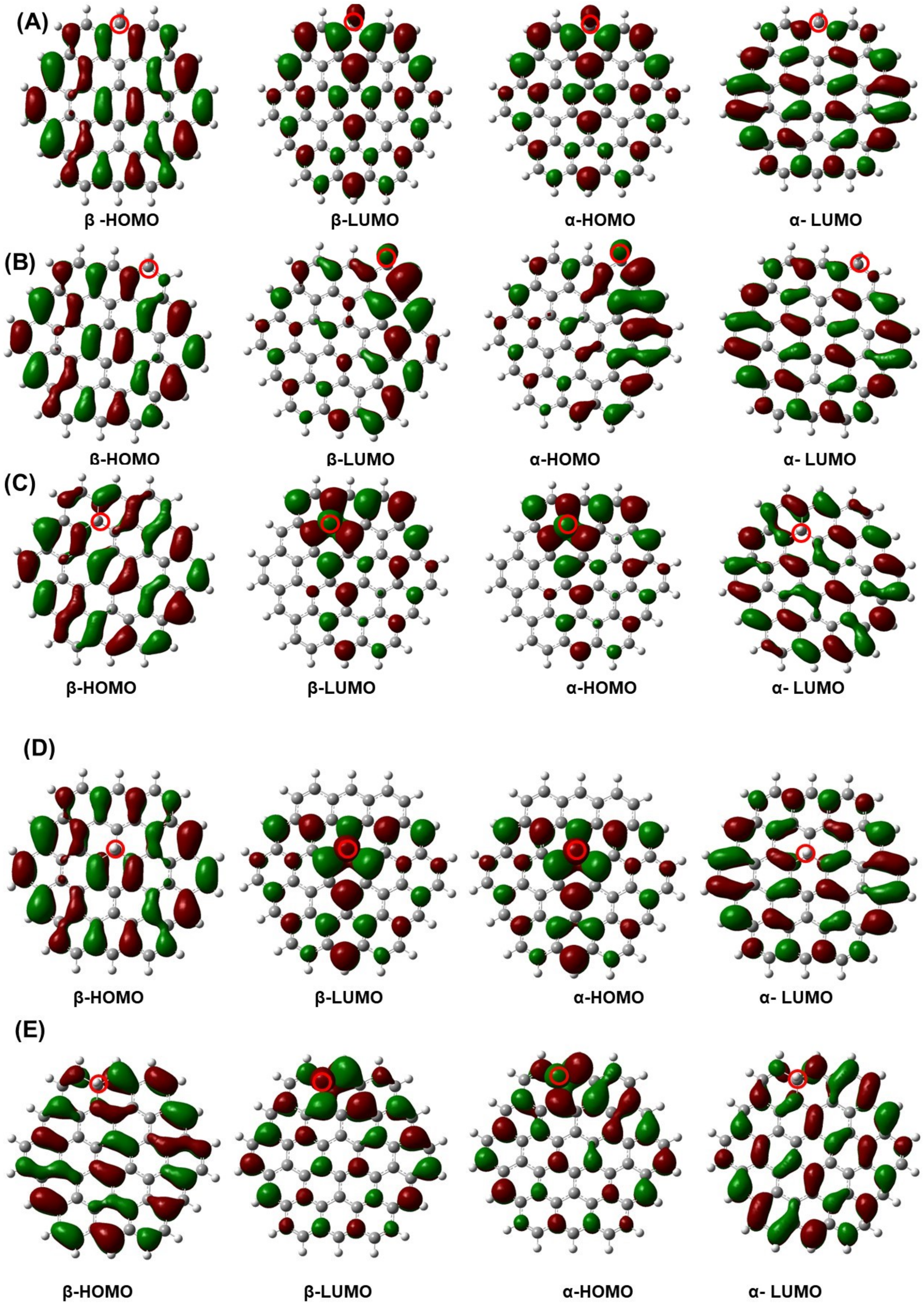

Figure S3: HOMO and LUMO plots of GQDs containing one sp^3 carbon (the sp^3 carbon is circled).

Table S3: Calculated excited states: absorption peaks and their principal electronic transitions for GQDs containing one sp^3 carbon at different positions. In this table and in the following tables, the date for the three key excited states are presented for each structure: the lowest-energy excitation, the second-lowest energy excitation, and the excitation with the largest oscillator strength in the region 300-500 nm.

Structures	Wavelength (nm)	Oscillator strength	Principal electronic transitions	S^2 value
A	1036	0.003	αHOMO - αLUMO (68%), βHOMO - βLUMO (32%)	0.81
	742	0.159	αHOMO - αLUMO (32%), βHOMO - βLUMO (65%)	0.83
	441	0.569	αHOMO-1 - αLUMO (42%), βHOMO - βLUMO+1 (40%)	0.85
B	843	0.002	αHOMO - αLUMO (61%), βHOMO - βLUMO (29%)	1.50
	656	0.105	αHOMO - αLUMO (28%), βHOMO - βLUMO (63%)	1.07
	393	0.492	αHOMO-2 - αLUMO+2 (34%), βHOMO-1 - βLUMO+2 (35%)	1.00
C	921	0.002	αHOMO - αLUMO (71%), βHOMO - βLUMO (25%)	0.88
	771	0.041	αHOMO - αLUMO (26%), βHOMO -βLUMO (60%)	0.83
	404	0.814	αHOMO-2 - αLUMO+1 (17%), αHOMO-1 - αLUMO+2 (18%)	0.83
D	971	0.001	αHOMO - αLUMO (64%), βHOMO - βLUMO (36%)	0.83
	787	0.065	αHOMO - αLUMO (36%), βHOMO - βLUMO (63%)	0.81
	395	0.220	βHOMO-1 - βLUMO+2 (22%), βHOMO - βLUMO+3 (39%)	1.72
E	994	0.001	αHOMO - αLUMO (62%), βHOMO - βLUMO (38%)	0.89
	738	0.111	αHOMO - αLUMO (31%), βHOMO - βLUMO (59%)	0.88
	440	0.354	αHOMO-1 - αLUMO (21%), βHOMO - βLUMO+1(28%)	1.08

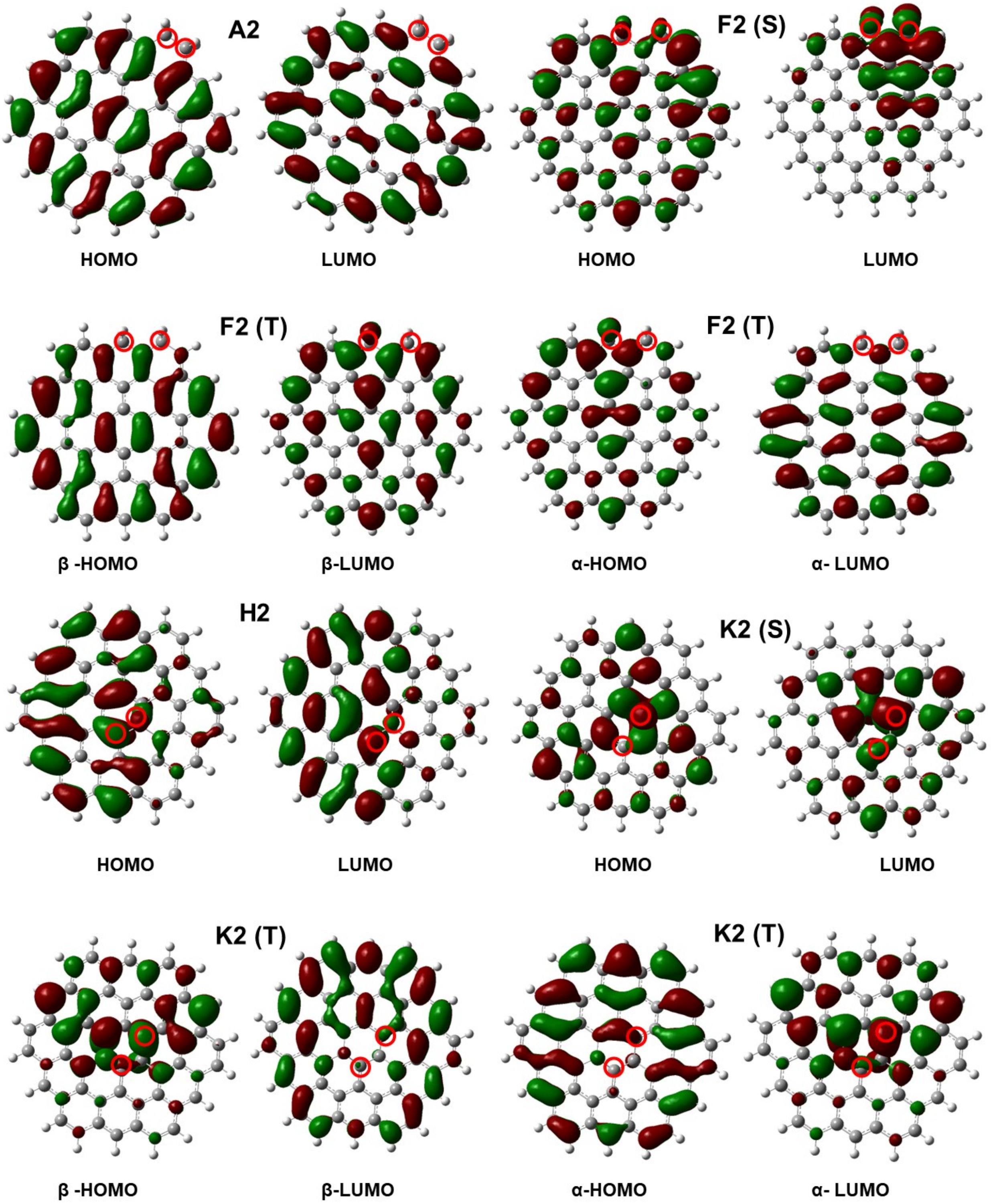
A2
HOMO
LUMO
F2 (S)
HOMO
LUMO
F2 (T)
β -HOMO
β-LUMO
F2 (T)
α-HOMO
α- LUMO
H2
HOMO
LUMO
K2 (S)
HOMO
LUMO
K2 (T)
β -HOMO
β-LUMO
K2 (T)
α-HOMO
α- LUMO

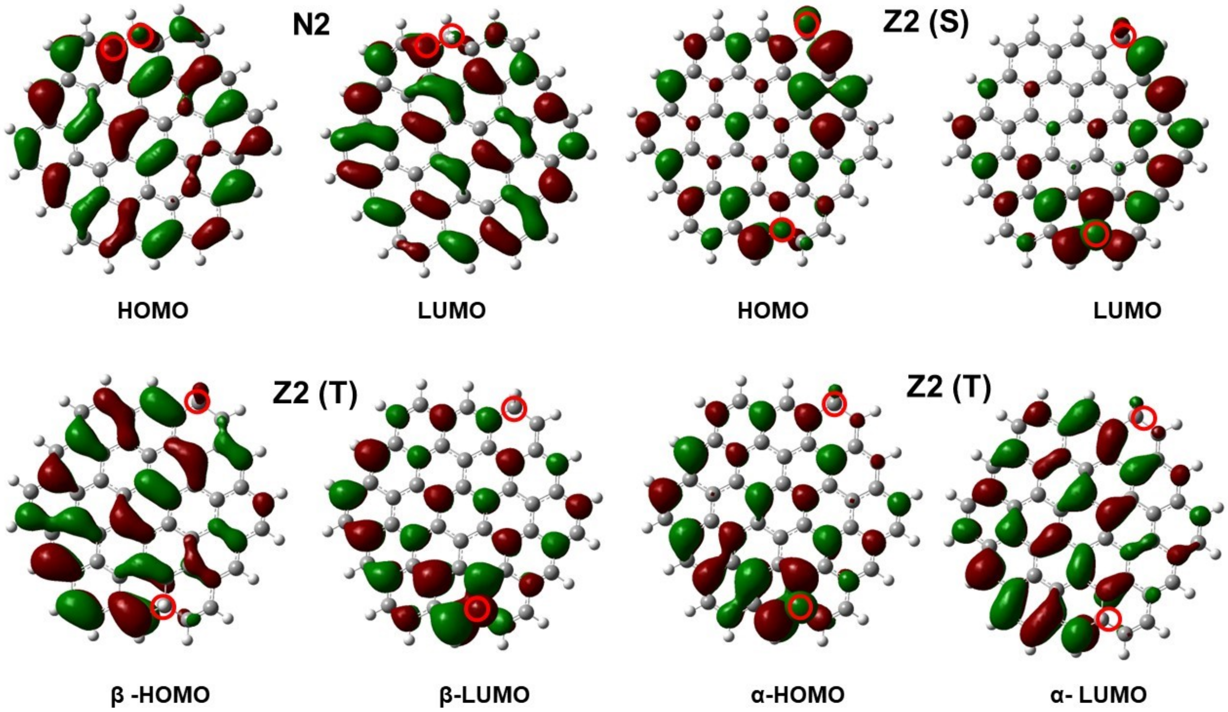

Figure S4: HOMO and LUMO plots of representative GQDs containing two sp^3 carbons: A2 - the most stable structure with two sp^3 carbons in edge positions; F2 - less stable structure with two sp^3 carbons in edge positions (singlet and triplet); H2 - the most stable structure with two sp^3 carbons in middle positions; K2 - less stable structure with two sp^3 carbons in middle positions (singlet and triplet); N2 - the most stable structure with two sp^3 carbons in edge and middle positions (the sp^3 carbons are circled); Z2 - less stable structure with two sp^3 carbons in edge and middle positions (singlet and triplet).

Table S4: Calculated excited states: absorption peaks and their principal electronic transitions for GQDs containing two sp^3 carbons at edge positions. The results for the most stable spin states are presented: singlets A2-D2 and triplets (T) E2-G2.

Structures	Wavelength (nm)	Oscillator strength	Principal electronic transition	S^2 value
A2	522	0.127	HOMO - LUMO (63%), HOMO-1 - LUMO+1 (17%)	0
	424	0.755	HOMO-1 - LUMO (52%), HOMO - LUMO+1 (48%)	
	414	0.348	HOMO-1 - LUMO+1 (35%), HOMO - LUMO+2 (49%)	
B2	625	0.243	HOMO - LUMO (98%), HOMO-1 - LUMO+1 (2%)	0
	456	0.081	HOMO - LUMO+2 (75%), HOMO - LUMO+3 (12%)	
	449	0.563	HOMO-1 - LUMO (48%), HOMO - LUMO+1 (33%)	
C2	657	0.338	HOMO - LUMO (92%), HOMO - LUMO (8%)	0
	498	0.054	HOMO - LUMO+2 (88%), HOMO-2 - LUMO (12%)	
	420	0.523	HOMO-1 - LUMO+1 (79%), HOMO-3 - LUMO+1 (10%)	
D2	655	0.217	HOMO - LUMO (62%), HOMO - LUMO+1 (28%)	0
	509	0.100	HOMO-2 - LUMO (84%), HOMO - LUMO+2 (12%)	
	366	0.975	HOMO-2 - LUMO+2 (66%), HOMO-1 - LUMO+1 (20%)	
E2 (T)	931	0.002	αHOMO - αLUMO (66%), βHOMO - βLUMO (33%)	2.22
	695	0.136	αHOMO - αLUMO (22%), βHOMO - βLUMO (45%)	2.21
	432	0.325	αHOMO-2 - αLUMO (27%), βHOMO - βLUMO+2 (15%)	2.31
F2 (T)	1159	0.003	αHOMO - αLUMO (65%), βHOMO - βLUMO (35%)	2.38
	788	0.228	αHOMO - αLUMO (34%), βHOMO - βLUMO (65%)	2.17
	367	0.359	αHOMO-3 - αLUMO (20%), βHOMO-2 - βLUMO+1 (36%)	2.19
G2 (T)	829	0.012	αHOMO - αLUMO (82%), βHOMO - βLUMO+1 (14%)	2.38
	629	0.088	αHOMO - αLUMO+1 (65%), βHOMO - βLUMO (15%)	2.22
	396	0.177	αHOMO - αLUMO+3 (16%), βHOMO-3 - βLUMO+1 (32%)	2.46

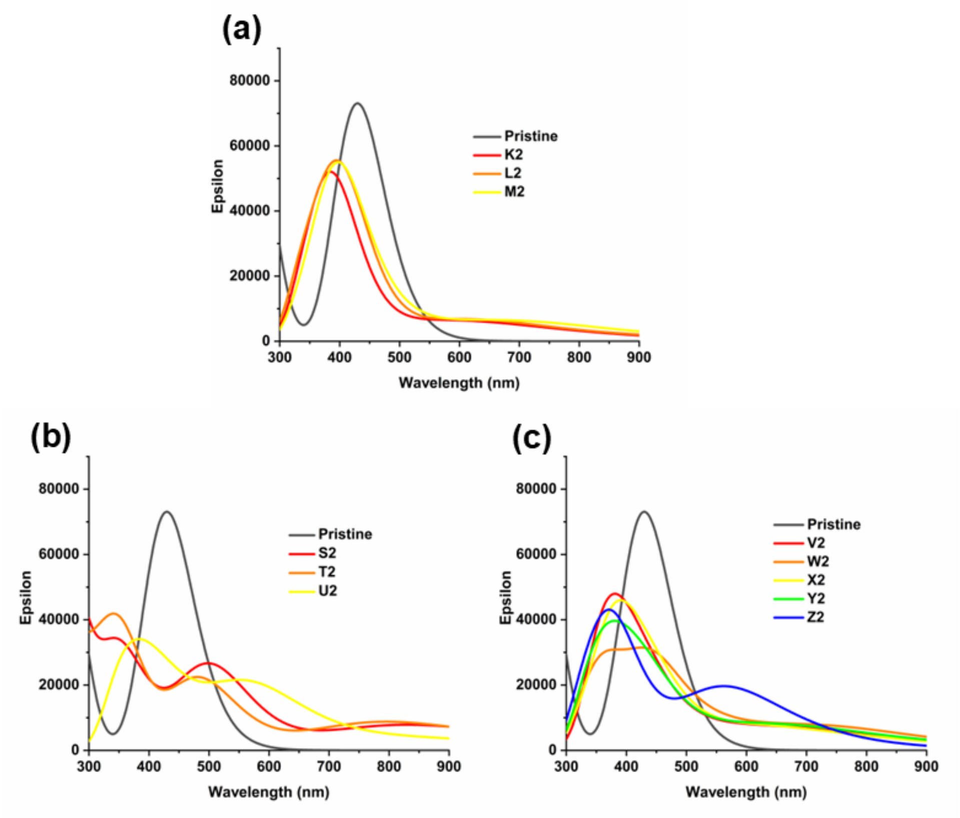

Figure S5: Calculated optical absorption spectra of GQDs containing two sp^3 carbons in the middle or middle and edge positions: (a) least stable structures with two sp^3 carbons in the middle of the GQD, separated by one sp^2 carbon, (b-c) least stable structures with sp^3 carbons in the middle and edge positions: (b) separated by 0, 2 or 4 sp^2 carbons, and (c) separated by 1, 3 and 9 sp^2 carbons. Spectra of the structures in their most stable spin states are shown: singlets for S2-T2, triplets for K2-M2, U2-Z2.

Table S5: Calculated excited states: absorption peaks and their principal electronic transitions for GQDs containing two sp^3 carbons at middle positions. The results for the most stable spin states are presented: singlets H2-J2 and triplets (T) K2-M2.

Structures	Wavelength (nm)	Oscillator strength	Principal electronic transition	S^2 value
H2	587	0.129	HOMO - LUMO (96%), HOMO-1 - LUMO+1 (4%)	0
	459	0.365	HOMO-1 - LUMO (56%), HOMO - LUMO+1 (38%)	
	416	0.212	HOMO-1 - LUMO+1 (82%), HOMO-2 - LUMO+2 (18%)	
I2	709	0.091	HOMO - LUMO (97%), HOMO - LUMO+2 (3%)	0
	510	0.062	HOMO-2 - LUMO (86%), HOMO - LUMO+2 (12%)	
	368	0.459	HOMO-2 - LUMO+2 (18%), HOMO-2 - LUMO+7 (27%)	
J2	966	0.035	HOMO - LUMO (100%)	0
	443	0.085	HOMO-1 - LUMO+1 (64%), HOMO - LUMO+6 (12%)	
	368	0.504	HOMO-2 - LUMO+2 (19%), HOMO-2 - LUMO+7 (18%)	
K2 (T)	862	0.003	αHOMO - αLUMO (44%), βHOMO - βLUMO (46%)	2.13
	651	0.032	αHOMO-1 - αLUMO (64%), αHOMO-1 - αLUMO+1 (18%)	2.15
	381	0.327	αHOMO-3 - αLUMO+1 (12%), βHOMO-6 - βLUMO (27%)	2.15
L2 (T)	963	0.004	αHOMO - αLUMO (79%), βHOMO - βLUMO (13%)	2.13
	713	0.037	αHOMO-1 - αLUMO (74%), βHOMO - βLUMO (15%)	2.23
	393	0.294	αHOMO-3 - αLUMO+1 (17%), βHOMO-1 - βLUMO+3 (14%)	2.29
M2 (T)	969	0.001	αHOMO - αLUMO (23%), βHOMO - βLUMO (63%)	2.13
	722	0.060	αHOMO-1 - αLUMO (76%), βHOMO - βLUMO+1 (17%)	2.17
	389	0.275	αHOMO-3 - αLUMO (11%), βHOMO-4 - βLUMO+1 (23%)	2.33

Table S6: Calculated excited states: absorption peaks and their principal electronic transitions for GQDs containing two sp^3 carbons at edge and middle positions. The results for the most stable spin states are presented: singlets N2-T2 and triplets (T) U2-Z2.

Structures	Wavelength (nm)	Oscillator strength	Principal electronic transition	S^2 value
N2	624	0.256	HOMO - LUMO (100%)	0
	439	0.732	HOMO - LUMO+2 (88%), HOMO - LUMO+3 (12%)	
	361	0.117	HOMO - LUMO+7 (49%), HOMO - LUMO+4 (12%)	
O2	544	0.001	HOMO - LUMO+1 (56%), HOMO-1 - LUMO (44%)	0
	472	0.438	HOMO-1 - LUMO (54%), HOMO - LUMO+1 (43%)	
	374	0.700	HOMO - LUMO+2 (47%), HOMO-2 - LUMO (41%)	
P2	625	0.141	HOMO - LUMO (100%)	0
	474	0.447	HOMO-1 - LUMO (49%), HOMO - LUMO+1 (41%)	
	370	0.484	HOMO-1 - LUMO+4 (27%), HOMO-1 - LUMO+2 (27%)	
Q2	535	0.111	HOMO - LUMO (92%), HOMO - LUMO+1 (4%)	0
	408	0.585	HOMO-1 - LUMO+1 (35%), HOMO-2 - LUMO (20%)	
	377	0.618	HOMO-2 - LUMO+2 (28%), HOMO-1 - LUMO+2 (23%)	
R2	810	0.085	HOMO - LUMO (100%)	0
	506	0.193	HOMO-1 - LUMO (55%), HOMO-2 - LUMO (27%)	
	381	0.273	HOMO - LUMO+5 (43%), HOMO-1 - LUMO+1 (34%)	
S2	837	0.190	HOMO - LUMO (100%)	0
	512	0.329	HOMO - LUMO+3 (39%), HOMO-1 - LUMO (31%)	
	493	0.240	HOMO - LUMO+3 (49%), HOMO-3 - LUMO (20%)	
T2	801	0.214	HOMO - LUMO (100%)	0
	541	0.045	HOMO-2 - LUMO (55%), HOMO-1 - LUMO (29%)	
	484	0.477	HOMO-1 - LUMO (28%), HOMO-2 - LUMO (27%)	
U2 (T)	1140	0.001	αHOMO - αLUMO (62%), βHOMO - βLUMO (38%)	2.17
	846	0.041	αHOMO - αLUMO (28%), βHOMO - βLUMO (29%)	2.10
	578	0.287	αHOMO-1 - αLUMO (21%), βHOMO-2 - βLUMO (28%)	2.18
V2 (T)	893	0.003	αHOMO - αLUMO (53%), βHOMO - βLUMO (47%)	2.12
	742	0.079	αHOMO - αLUMO (46%), βHOMO - βLUMO (45%)	2.19
	432	0.211	αHOMO-2 - αLUMO (24%), βHOMO - βLUMO+2 (32%)	2.19
W2 (T)	1027	0.002	αHOMO - αLUMO (81%), βHOMO - βLUMO (16%),	2.10
	542	0.079	αHOMO-1 - αLUMO+1 (42%), βHOMO-1 - βLUMO (33%)	2.15
	440	0.350	αHOMO-2 - αLUMO (35%), βHOMO - βLUMO+2 (29%)	2.22
X2 (T)	933	0.004	αHOMO - αLUMO (80%), βHOMO - βLUMO (19%)	2.11
	647	0.041	αHOMO-1 - αLUMO (27%), βHOMO - βLUMO+1 (34%)	2.70
	434	0.265	αHOMO-2 - αLUMO (31%), βHOMO - βLUMO+2 (19%)	2.42
Y2 (T)	960	0.005	αHOMO - αLUMO (80%), βHOMO - βLUMO (20%)	2.14
	700	0.056	αHOMO-1 - αLUMO (27%), βHOMO - βLUMO (24%)	2.22
	353	0.208	αHOMO-3 - αLUMO+1 (14%), αHOMO-1 - αLUMO+3 (19%)	2.32
Z2 (T)	867	0.004	αHOMO - αLUMO (74%), βHOMO - βLUMO (26%)	2.11
	642	0.047	αHOMO-1 - αLUMO+1 (18%), βHOMO-1 - βLUMO+1 (31%)	2.22
	387	0.151	αHOMO-1 - αLUMO+1 (28%), βHOMO-1 - βLUMO+1 (35%)	2.35

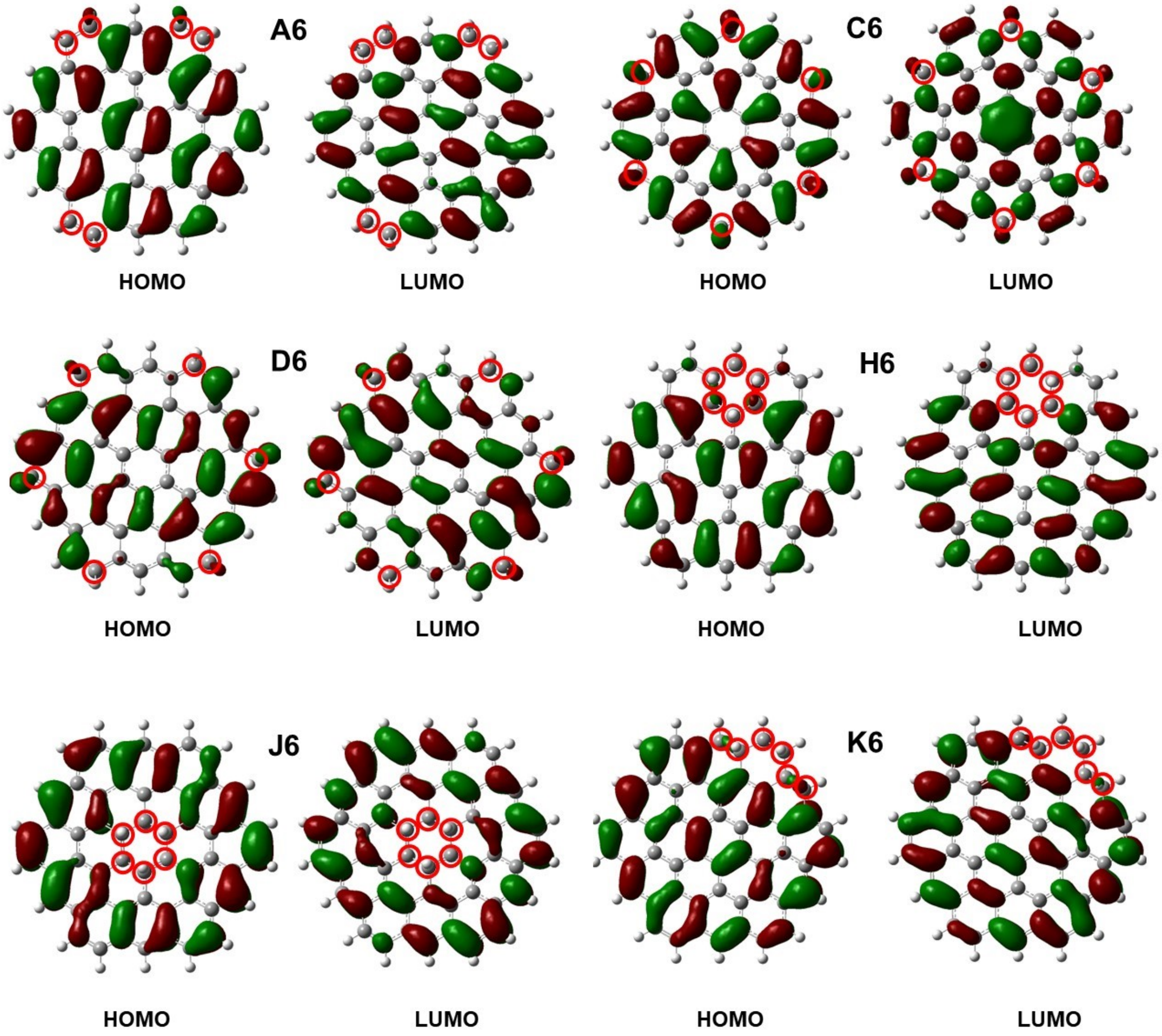

Figure S6: HOMO and LUMO plots of representative GQDs containing six sp^3 carbons: A6 - dimers of sp^3 carbons along the edge of the GQD; C6, D6 - isolated sp^3 carbons along the edge of the GQD; H6 - six-membered ring of sp^3 carbons; K6 - chain of sp^3 carbons along the edge of the GQD (the sp^3 carbons are circled).

Table S7: Calculated excited states: absorption peaks and their principal electronic transitions for GQDs containing six sp^3 carbons. The results for the most stable spin states are presented: singlets A6-D6, H6-M6 and triplets (T) E6-G6.

Structures	Wavelength (nm)	Oscillator strength	Principal electronic transition	S^2 value
A6	515	0.250	HOMO - LUMO (94%), HOMO-1 - LUMO+1 (6%)	0
	416	0.180	HOMO - LUMO+2 (61%), HOMO-1 - LUMO (19%)	
	407	0.468	HOMO-1 - LUMO (34%), HOMO - LUMO+2 (35%)	
B6	515	0.008	HOMO-1 - LUMO (45%), HOMO - LUMO+1 (55%)	0
	492	0.017	HOMO - LUMO (72%), HOMO-1 - LUMO+1 (28%)	
	431	0.541	HOMO-1 - LUMO (52%), HOMO - LUMO+1 (45%)	
C6	390	0.632	HOMO-2 - LUMO+1 (50%), HOMO-1 - LUMO+2 (50%)	0
	345	0.086	HOMO - LUMO+3 (93%), HOMO-1 - LUMO+5 (4%)	
	307	0.032	HOMO-3 - LUMO+6 (45%), HOMO-2 - LUMO+5 (46%)	
D6	658	0.424	HOMO-1 - LUMO (44%), HOMO - LUMO+1 (44%)	0
	413	0.027	HOMO-2 - LUMO+3 (100%)	
	373	0.033	HOMO-3 - LUMO+2 (97%), HOMO - LUMO+6 (3%)	
E6 (T)	1142	0.052	αHOMO - αLUMO (47%), βHOMO - βLUMO+1 (44%)	2.14
	870	0.153	αHOMO-2 - αLUMO (46%), βHOMO - βLUMO+2 (33%)	2.42
	383	0.179	αHOMO-3 - αLUMO+1 (22%), βHOMO-1 - βLUMO+3 (30%)	2.42
F6 (T)	1176	0.004	αHOMO - αLUMO (63%), βHOMO - βLUMO (33%)	2.28
	865	0.061	αHOMO - αLUMO (37%), βHOMO - βLUMO+3 (33%)	2.72
	479	0.202	αHOMO-2 - αLUMO+1 (33%), βHOMO-1 - βLUMO+2 (17%)	2.43
G6 (T)	1292	0.021	αHOMO - αLUMO+1 (25%), βHOMO-1 - βLUMO (72%)	2.07
	925	0.025	αHOMO-1 - αLUMO (20%), βHOMO - βLUMO+1 (44%)	2.08
	525	0.265	αHOMO-3 - αLUMO+1 (24%), αHOMO-3 - αLUMO+2 (15%)	2.49
H6	511	0.001	HOMO-1 - LUMO (48%), HOMO - LUMO+1 (52%)	0
	430	0.654	HOMO-1 - LUMO (51%), HOMO - LUMO+1 (47%)	
	418	0.328	HOMO - LUMO (35%), HOMO-1 - LUMO+1 (65%)	
I6	640	0.253	HOMO - LUMO (100%)	0
	495	0.018	HOMO - LUMO+2 (75%), HOMO-2 - LUMO (15%)	
	431	0.545	HOMO-1 - LUMO (33%), HOMO-2 - LUMO (27%)	
J6	377	0.238	HOMO - LUMO (29%), HOMO-1 - LUMO+1 (29%)	0
	354	0.646	HOMO-3 - LUMO+2 (32%), HOMO-2 - LUMO+3 (32%)	
	269	0.879	HOMO-6 - LUMO+3 (49%), HOMO-3 - LUMO+6 (34%)	
K6	641	0.370	HOMO - LUMO (100%)	0
	427	0.297	HOMO-2 - LUMO (43%), HOMO - LUMO+1 (28%)	
	289	0.576	HOMO-3 - LUMO+3 (70%), HOMO-2 - LUMO+2 (15%)	
L6	745	0.264	HOMO - LUMO (98%)	0
	501	0.086	HOMO-2 - LUMO (53%), HOMO-1 - LUMO (27%)	
	458	0.478	HOMO-2 - LUMO (28%), HOMO - LUMO+1 (27%)	
M6	875	0.184	HOMO - LUMO (97%)	0
	657	0.002	HOMO - LUMO+1 (55%), HOMO-1 - LUMO (45%)	
	525	0.417	HOMO-1 - LUMO (51%), HOMO - LUMO+1 (42%)	

Table S8: Relative energies, formation energies, HOMO and LUMO energies and band gaps of triangular GQDs structures: fully sp^2 structures TZ (with zigzag edges) and TA (with armchair edges), and structures TZ-A6, TZ-B6, TA-A6, TA-B6 and TA-C6 containing six sp^3 carbons at various positions. The stable spin states for TA and TA-A6 are singlets, and the stable spin states for TA-B6, TA-C6 and all TZ structures are triplets.

Structure	Relative energy (eV)	Formation energy (eV/1C(sp^3))	αHOMO, βHOMO (eV)	αLUMO, βLUMO (eV)	αBand gap, βBand gap (eV)
TZ (T)	-	-	-4.48, -4.41	-3.19, -3.11	1.28, 1.30
TZ-A6 (T)	0.00	0.08	-4.29, -4.36	-2.52, -2.67	1.78, 1.70
TZ-B6 (T)	0.66	0.19	-4.49, -4.37	-2.89, -2.81	1.60, 1.56
TA (S)	-	-	-5.32	-2.13	3.19
TA-A6 (S)	0.00	-0.04	-4.59	-2.59	2.00
TA-B6 (T)	1.92	0.28	-4.42, -5.37	-1.96, -2.82	2.46, 2.54
TA-C6 (T)	2.31	0.34	-4.66, -5.27	-2.05, -2.66	2.62, 2.61

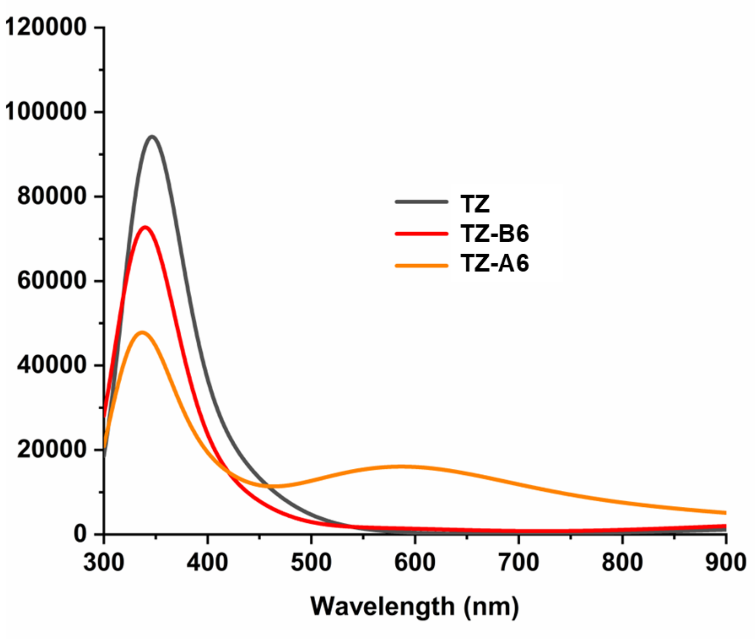

Figure S7: Calculated optical absorption spectra of triangular GQDs with zigzag edges. TZ: fully sp^2 structure; TZ-A6: 6 sp^3 carbons extending from the edge into the middle of the GQD; TZ-B6: 6 sp^3 carbons along the zigzag edge.

.

Bibliography

(1) B. Shi, D. Nachtigallová, A. J. Aquino, F. B. Machado and H. Lischka, “High-level theoretical benchmark investigations of the UV-vis absorption spectra of paradigmatic polycyclic aromatic hydrocarbons as models for graphene quantum dots”, *The Journal of Chemical Physics*, 2019, **150**, 124302.

(2) I. J. Webster, J. L. Beckham, N. D. Johnson and M. A. Duncan, “Photochemical Synthesis and Spectroscopy of Covalent PAH Dimers”, *The Journal of Physical Chemistry A*, 2022, **126**, 1144–1157.

(3) J. W. Patterson, “The ultraviolet absorption spectra of coronene”, *Journal of the American Chemical Society*, 1942, **64**, 1485–1486.
